# Supplementary material for: Evolution of intraocular pressure after cataract surgery in nonglaucomatous patients: A post-hoc analysis of PERCEPOLIS clinical trial data
Source: PLoS One. 2026 May 19;21(5):e0349310. doi: 10.1371/journal.pone.0349310 (PMC13186369; doi:10.1371/journal.pone.0349310)
Supplement: S1 Fig — All studies used phacoemulsification to remove the lens [13–21,23–25,27,29–35]. Our data are shown in red. IOP, intraocular pressure; m, months. (DOCX) [file pone.0349310.s001.docx]

## S1 Fig Previous s**tudies showing absolute (mmHg) (A) and % (B) change in IOP over time in nonglaucomatous patients after cataract surgery.** All studies used phacoemulsification to remove the lens [13–21,23–25,27,29–35]**.** Our data are shown in red. IOP, intraocular pressure; m, months.


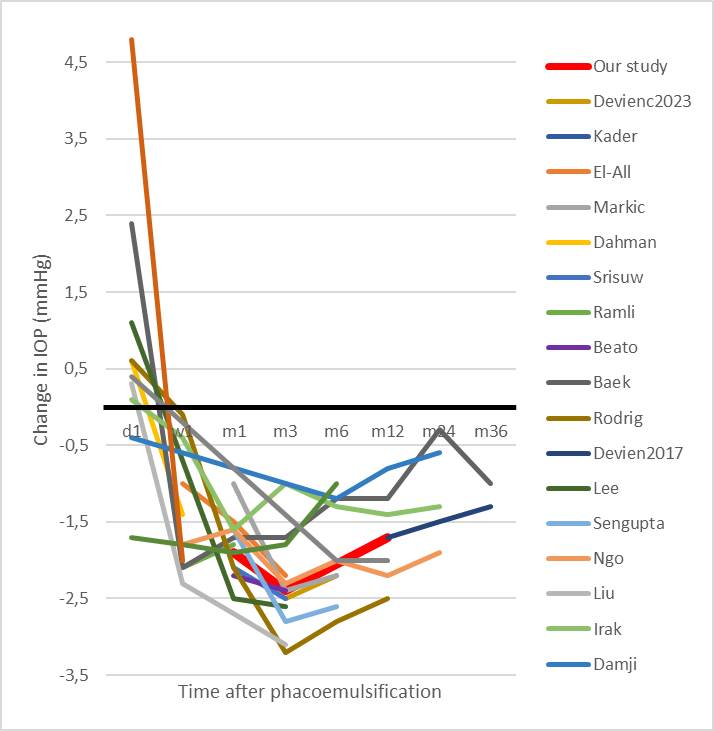

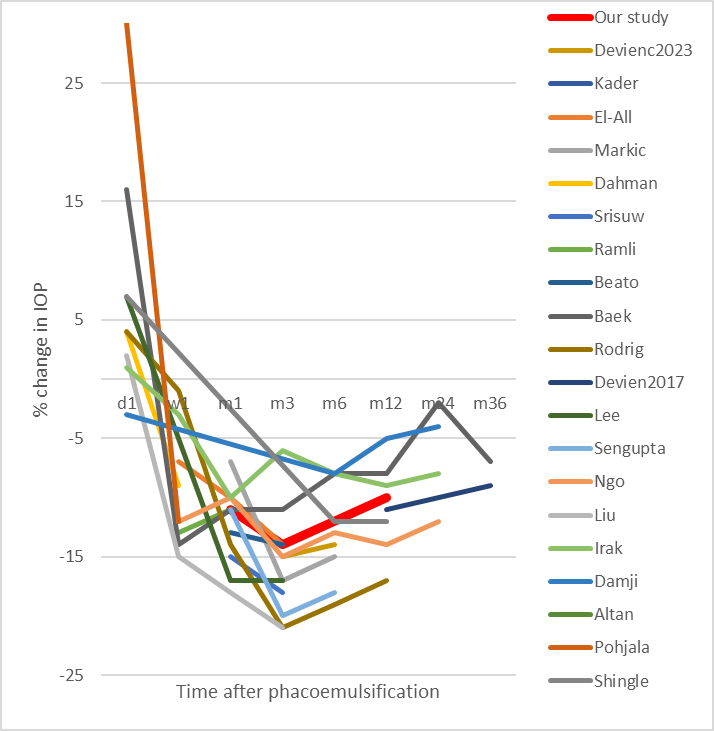


A: Absolute IOP change

B: % IOP change
