# Supplementary material for: Evolution of intraocular pressure after cataract surgery in nonglaucomatous patients: A post-hoc analysis of PERCEPOLIS clinical trial data
Source: PLoS One. 2026 May 19;21(5):e0349310. doi: 10.1371/journal.pone.0349310 (PMC13186369; doi:10.1371/journal.pone.0349310)
Supplement: S2 Fig — The three preoperative IOP subgroups are indicated by color. On Pearson correlation coefficient analysis, r = −0.60 [−0.67; −0.51], p < 0.0001. M, month; IOP, intraocular pressure. (DOCX) [file pone.0349310.s002.docx]

## S2 Fig Relationship between preoperative and 3-month postoperative IOP (mmHg) (*n*=241). The three preoperative IOP subgroups are indicated by color. On Pearson correlation coefficient analysis, r=-0.60 [-0.67; -0.51], p<0.0001. M, month; IOP, intraocular pressure.
