# Supplementary material for: Evolution of intraocular pressure after cataract surgery in nonglaucomatous patients: A post-hoc analysis of PERCEPOLIS clinical trial data
Source: PLoS One. 2026 May 19;21(5):e0349310. doi: 10.1371/journal.pone.0349310 (PMC13186369; doi:10.1371/journal.pone.0349310)

## S3 Fig Absolute IOP change (mmHg) over time in the nonglaucomatous and glaucomatous groups in previous studies that compared the two groups directly after cataract surgery. Cataract surgery was conducted with phacoemulsification in all studies [14,21,27–29,31,34] except Shah et al., who used femtosecond-laser assisted cataract surgery [80]. The data of Coh et al. were not added because they only studied a single timepoint (4 months: nonglaucomatous eyes changed from 14.7 to 11.9 mmHg; POAG eyes changed from 14.9 to 12.2 mmHg) [41]. The data of Shingleton et al. (2006) were not added because they only studied two timepoints that were out of the x-axis range (nonglaucomatous eyes changed from 15.9 to 14.2 and 14.4 mmHg at 3 and 5 years; glaucomatous eyes changed from 18.4 to 17.0 and 16.6 mmHg at 3 and 5 years) [22]. Our data from nonglaucomatous patients were added to both graphs for comparison (red). d, day; GC, glaucoma; IOP, intraocular pressure; m, month; NG, nonglaucomatous; PACG, primary angle open glaucoma; POAG, primary open angle glaucoma; PXFGC, pseudoexfoliation glaucoma; w, week.


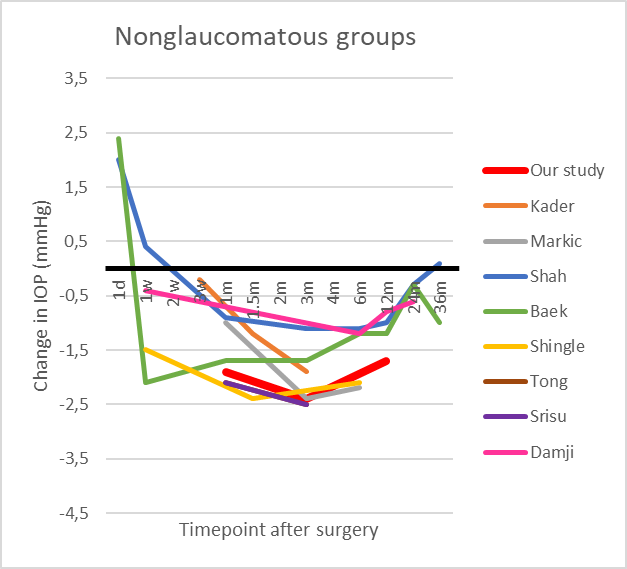

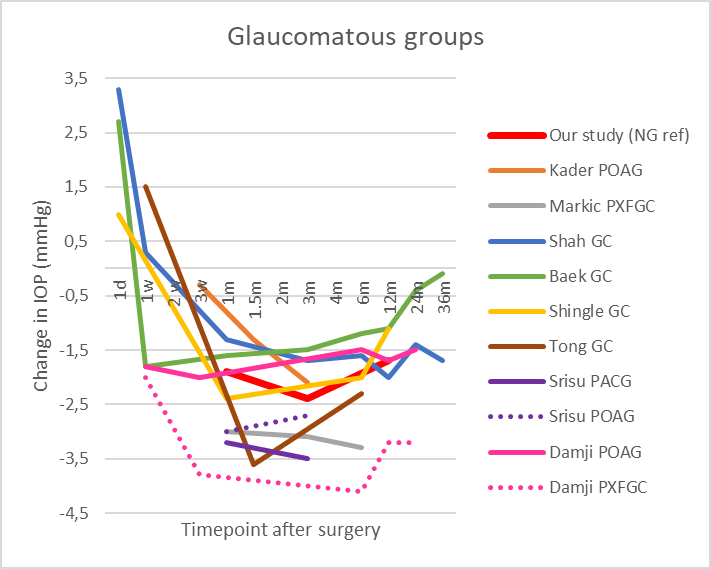

Supplement: S3 Fig — Cataract surgery was conducted with phacoemulsification in all studies [14,21,27–29,31,34] except Shah et al., who used femtosecond-laser assisted cataract surgery [81]. The data of Coh et al. were not added because they only studied a single timepoint (4 months: nonglaucomatous eyes changed from 14.7 to 11.9 mmHg; POAG eyes changed from 14.9 to 12.2 mmHg) [41]. The data of Shingleton et al. (2006) were not added because they only studied two timepoints that were out of the x-axis range (nonglaucomatous eyes changed from 15.9 to 14.2 and 14.4 mmHg at 3 and 5 years; glaucomatous eyes changed from 18.4 to 17.0 and 16.6 mmHg at 3 and 5 years) [22]. Our data from nonglaucomatous patients were added to both graphs for comparison (red). d, day; GC, glaucoma; IOP, intraocular pressure; m, month; NG, nonglaucomatous; PACG, primary angle open glaucoma; POAG, primary open angle glaucoma; PXFGC, pseudoexfoliation glaucoma; w, week. (DOCX) [file pone.0349310.s003.docx]
