# Supplementary material for: Evolution of intraocular pressure after cataract surgery in nonglaucomatous patients: A post-hoc analysis of PERCEPOLIS clinical trial data
Source: PLoS One. 2026 May 19;21(5):e0349310. doi: 10.1371/journal.pone.0349310 (PMC13186369; doi:10.1371/journal.pone.0349310)
Supplement: S4 Fig — The details of the studies are summarized in S10 Table. (A) Two studies involved multiple preoperative IOP measurements and also had a control group that resembled the cataract-surgery group but did not undergo cataract surgery [6,84]. (B) Five studies involved multiple preoperative IOP measurements only [6,29,32,49,84]. (C) Five studies used the contralateral unoperated eye [46,47,83,91,92]. The legends show the glaucoma status of the eyes, and their preoperative IOP in brackets. CS, cataract surgery; GC, glaucoma; NG, nonglaucomatous; OHT, ocular hypertension, PACG, primary angle-closure glaucoma; preop, preoperative. (DOCX) [file pone.0349310.s004.docx]

## S4 Fig. IOP change in studies that controlled for RTM. The details of the studies are summarized in S10 Table. (A) Two studies involved multiple preoperative IOP measurements and also had a control group that resembled the cataract-surgery group but did not undergo cataract surgery [6,84]. (B) Five studies involved multiple preoperative IOP measurements only [6,29,32,49,84]. (C) Five studies used the contralateral unoperated eye [46,47,83,91,92]. The legends show the glaucoma status of the eyes, and their preoperative IOP in brackets. CS, cataract surgery; GC, glaucoma; NG, nonglaucomatous; OHT, ocular hypertension, PACG, primary angle-closure glaucoma; preop, preoperative.


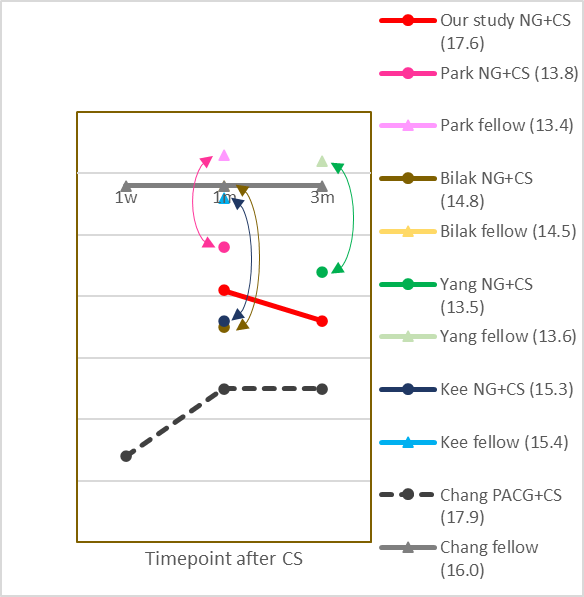

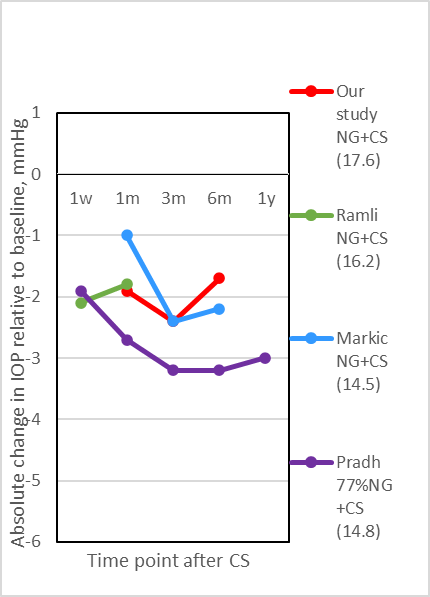

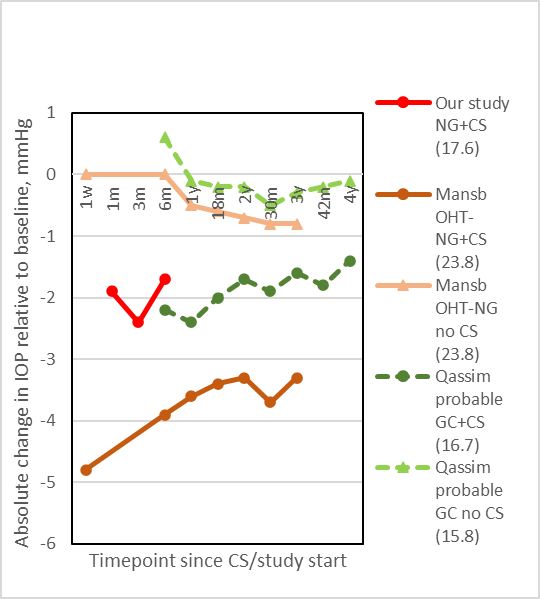


(B) Multiple preop IOP values

(A) Multiple preop IOP values + control group

(C) Contralateral eye control
