# Supplementary material for: Evolution of intraocular pressure after cataract surgery in nonglaucomatous patients: A post-hoc analysis of PERCEPOLIS clinical trial data
Source: PLoS One. 2026 May 19;21(5):e0349310. doi: 10.1371/journal.pone.0349310 (PMC13186369; doi:10.1371/journal.pone.0349310)
Supplement: S1 Table — (DOCX) [file pone.0349310.s005.docx]

### S1 Table. Previous studies examining the effect of phacoemulsification cataract surgery on IOP in nonglaucomatous eyes over time

| Study year country^Ref^ | Study design (study period) [angle type] | No. eyes/ patients | Age, y | Mean baseline IOP,or category (mean), mmHg | Follow-up | Absolute IOP change, mmHg | % IOP change |
| --- | --- | --- | --- | --- | --- | --- | --- |
| Our study France | RCT: subluxation vs DAC (2015–2016) | 238/238 | 74 | 17.6 | 1 m | -1.9 | -11% |
|  |  | 241/241 |  |  | 3 m | -2.4 | -14% |
|  |  | 174/174 |  |  | 1 y | -1.7 | -10% |
| Devience 2023 USA[13] | Pros (period?) | 90/90 | 67 | 16.1 | 3 m | -2.5 | -15% |
|  |  |  |  |  | 6 m | -2.2 | -12% |
| Kader 2022 India[14] | Pros (2016–?) | 51/51 | 69 | 16.5 | 3 w | -0.2 | -1% |
|  |  |  |  |  | 6 w | -1.2 | -7% |
|  |  |  |  |  | 3 m | -1.9 | -11% |
| El-All 2022 Egypt[25] | Retr (2018–2020) [no angle defects] | 100/100 | 63 | 15.3 | 1 w | -1.0 | -7% |
|  |  |  |  |  | 1 m | -1.5 | -10% |
|  |  |  |  |  | 3 m | -2.2 | -14% |
| Markic 2022 Bosnia[29] | Pros (2016–2018) [all OA] | 31/31 | 71 | 14.5 | 1 m | -1.0 | -7% |
|  |  |  |  |  | 3 m | -2.4 | -17% |
|  |  |  |  |  | 6 m | -2.2 | -15% |
| Dhamankar 2020 India[30] | Retr (period?) | 105/82 | 60 | 15.1 | 1 d | +0.6 | +4% |
|  |  |  |  |  | 1 w | -1.4 | -9% |
|  |  |  |  |  | 3 w | -1.6 | -11% |
| Srisuwanporn 2020 Thailand[78] | Retr (2011–2015) | 86/86 | 70 | 13.6 | 1 m | -2.1 | -15% |
|  |  |  |  |  | 3 m | -2.5 | -18% |
| Ramli 2019 Malaysia[32] | Pros (period?) | 86/86 | 64 | 16.2 | 1 w | -2.1 | -13% |
|  |  |  |  |  | 1 m | -1.8 | -11% |
| Beato 2019 Portugal[33] | Pros: DM vs noDM (2015–2016) [all OA] | 89:  45/45 DM  44/44 noDM | 73 DM  71 noDM | 17.8 DM  16.9 noDM | 1 m | -1.7 DM  -2.2 noDM | -10% DM  -13% noDM |
|  |  |  |  |  | 6 m | -2.9 DM  -2.4 noDM | -16% DM  -14% noDM |
| Baek 2019 Korea[34] | Retr (2018–?) | 648/648 | 65 | 15.0 | 1 d | +2.4 | +16% |
|  |  |  |  |  | 1 w | -2.1 | -14% |
|  |  |  |  |  | 1 m | -1.7 | -11% |
|  |  |  |  |  | 3 m | -1.7 | -11% |
|  |  |  |  |  | 6 m | -1.2 | -8% |
|  |  |  |  |  | 1 y | -1.2 | -8% |
|  |  |  |  |  | 2 y | -0.3 | -2% |
|  |  |  |  |  | 3 y | -1.0 | -7% |
| Rodrigues 2018 Brazil[35] | Retr (2012–2016) [all OA] | 231/179 | 72 | 15.1 | 1 d | +0.6 | +4% |
|  |  |  |  |  | 7 d | -0.1 | -1% |
|  |  |  |  |  | 2 w | -1.1 | -7% |
|  |  |  |  |  | 1 m | -2.1 | -14% |
|  |  |  |  |  | 3 m | -3.2 | -21% |
|  |  |  |  |  | 6 m | -2.8 | -19% |
|  |  |  |  |  | 1 y | -2.5 | -17% |
| DeVience 2017 USA[15] | Retr (2006–2008) | 115/115 | 70 | 14.9 | 1 y | -1.7 | -11% |
|  |  |  |  |  | 2 y | -1.5 | -10% |
|  |  |  |  |  | 3 y | -1.3 | -9% |
| Lee 2016 USA[16] | Pros (2009–2012) | 161/116 | 75 | 14.9 | 1 d | +1.1 | +7% |
|  |  |  |  |  | 1 m | -2.5 | -17% |
|  |  |  |  |  | 3 m | -2.6 | -17% |
| Sengupta 2016 India[17] | RCT: CPS vs MICS [all OA] | 250/250 CPS | 58 | 14.2 | 1 m | -1.6 | -11% |
|  |  |  |  |  | 3 m | -2.8 | -20% |
|  |  |  |  |  | 6 m | -2.6 | -18% |
| Ngo 2016 Singapore[18] | Retr (2007–2008) | 116/116 | 71 | 15.4 | 1 w | -1.8 | -12% |
|  |  |  |  |  | 1 m | -1.6 | -10% |
|  |  |  |  |  | 3 m | -2.3 | -15% |
|  |  |  |  |  | 6 m | -2.0 | -13% |
|  |  |  |  |  | 1 y | -2.2 | -14% |
|  |  |  |  |  | 2 y | -1.9 | -12% |
| Liu 2013 China[19] | Retr (2005–2007) | 361 | - | 14.9 | 1 d | +0.3 | +2% |
|  |  |  |  |  | 1 w | -2.3 | -15% |
|  |  |  |  |  | 1 m | -2.7 | -18% |
|  |  |  |  |  | 3 m | -3.1 | -21% |
| Irak 2010 USA[20] | Retr (1999) | 266/266 | 71 | 15.8 |  |  |  |
|  |  | 251 |  |  | 1 d | +0.1 | +1% |
|  |  | 197 |  |  | 1 w | -0.4 | -3% |
|  |  | 186 |  |  | 1 m | -1.6 | -10% |
|  |  | 141 |  |  | 3 m | -1.0 | -6% |
|  |  | 132 |  |  | 6 m | -1.3 | -8% |
|  |  | 168 |  |  | 1 y | -1.4 | -9% |
|  |  | 139 |  |  | 2 y | -1.3 | -8% |
| Damji 2006 Canada[21] | Pros (?) | 83/83 | 73 | 15.2 |  |  |  |
|  |  | 55 |  |  | 1 w | -0.4 | -3% |
|  |  | 65 |  |  | 3 w | -0.6 | -4% |
|  |  | 40 |  |  | 6 w | -1.4 | -9% |
|  |  | 48 |  |  | 6 m | -1.2 | -8% |
|  |  | 55 |  |  | 1 y | -0.8 | -5% |
|  |  | 58 |  |  | 2 y | -0.6 | -4% |
| Shingleton 2006 USA[22] | Retr (1995-1997) | 59/59 | 69 | 15.9 | 3 y | -1.7 | -11% |
|  |  |  |  |  | 5 y | -1.5 | -9% |
| Altan 2004 Turkey[23] | Pros (2001) [all OA] | 53/49 | 67 | 15.1 | 1 d | -1.7 | -11% |
|  |  |  |  |  | 1 w | -1.8 | -12% |
|  |  |  |  |  | 1 m | -1.9 | -13% |
|  |  |  |  |  | 3 m | -1.8 | -12% |
|  |  |  |  |  | 6 m | -1.0 | -7% |
| Pohjalainen 2001 Finland[24] | Retr (1995) | 137/? | 67 | 16.2 | 1 d | +4.8 | +30% |
|  |  |  |  |  | 1 w | -2.0 | -12% |
|  |  |  |  |  | 4 m | -3.4 | -21% |
|  |  |  |  |  | 1-2.7 y | -3.9 | -24% |
| Shingleton 1999 USA[27] | Retr (1995–1997) | 164/143 | 72 | 16.4 | 1 d | +0.4 | +2% |
|  |  |  |  |  | 3-6 w | -1.1 | -7% |
|  |  |  |  |  | 6 m | -2.0 | -12% |
|  |  |  |  |  | 1 y | -2.0 | -12% |

CPS, conventional phacoemulsification surgery; DAC, divide-and-conquer cataract extraction; DM, diabetes mellitus; IOP, intraocular pressure; m, months; MICS, manual small-incision surgery; OA, open angle; pros, prospective; RCT, randomized controlled trial; Retr, retrospective; w, week; y, year.
