# Supplementary material for: Evolution of intraocular pressure after cataract surgery in nonglaucomatous patients: A post-hoc analysis of PERCEPOLIS clinical trial data
Source: PLoS One. 2026 May 19;21(5):e0349310. doi: 10.1371/journal.pone.0349310 (PMC13186369; doi:10.1371/journal.pone.0349310)
Supplement: S2 Table — (DOCX) [file pone.0349310.s006.docx]

### S2 Table. Previous studies that searched for pre/perioperative factors that associate with greater IOP change in nonglaucomatous eyes

| Study and year | No. eyes/patients, study type | Postoperative time | Type of patient | Type analysis and way IOP was expressed | Greater in older or younger | Higher in sex indicated | Cataract density | Higher preoperative IOP | Smaller preoperative ACD | Smaller preoperative AXL | Greater LT | Smaller preoperative LP | Smaller preoperative RLP | Implant power | EPT, CDE, phacotime, or use of ultrasound |
| --- | --- | --- | --- | --- | --- | --- | --- | --- | --- | --- | --- | --- | --- | --- | --- |
| Our study | 241/241, RCT | 3m | NG | UV AC | NS | NS | NS | 0.001 | NS | NS | NS |  |  | NS | EPT NS |
|  |  |  |  | MV AC | 0.04 old | 0.01M | NS | 0.001 | NS | NS | NS |  |  | NS | EPT NS |
| Uzun 2025[58] | 171/145, ret | 3m | NG | UV AC |  |  |  | r=0.482 | 0.034 | NS | 0.026 |  |  |  |  |
|  |  |  |  | MV AC |  |  |  | 0.001 | 0.009 |  | 0.001 |  |  |  |  |
| Dawoo 2023[57] | 42/42, pros | 2m | NG | UV AC | NS |  |  | r=0.58 |  |  |  |  |  |  |  |
| DeVienc 2023[13] | 90/90, pros | 6m | NG | UV %C | NS |  |  | <0.001 |  |  |  |  |  |  | CDE & FLACS NS |
| Kader 2022[14] | 51/51, pros | 3m | NG | UV%C |  |  |  |  |  |  | NS |  |  |  |  |
| El-All 2022[25] | 100/100, ret | 1w-3m | NG no PACS | UV A^iop^ | NS |  |  |  |  |  |  |  |  |  |  |
| Markic 2022[29] | 31/31, pros | 6m | NG OA | UV AC | NS | NS |  | 0.01 | NS | NS | NS | NS | NS |  |  |
|  |  |  |  | UV %C | NS | NS |  | NS | NS | NS | NS | NS | NS |  |  |
| Tayyab 2021[38] | 139/139, pros | 1m | NG | UV AC | NS | NS |  |  |  |  |  |  |  |  |  |
| Ramez 2021[39] | 31/31, pros | 3m | NG | UV AC |  |  |  | <0.01 | NS |  |  |  |  |  | CDE NS |
| Ramli 2019[32] | 86/86, pros | 1m | NG | UV AC | NS | NS |  | r=0.54 | NS | 0.01 | 0.09 |  |  |  |  |
|  |  |  |  | MV AC | NS | NS |  | 0.001 |  | 0.018 | NS |  |  |  |  |
| Beato 2019[33] | 44/44, pros | 6m | NG OA | UV AC | NS | NS | NS | 0.001 |  | NS |  |  |  |  | CDE NS |
|  |  |  |  | MV AC | NS | NS | NS | 0.003 |  | NS |  |  |  |  | CDE NS |
| Baek 2019[34] | 754/754, ret | 1y | 14% GC, 77% NG no PACS | UV AC | 0.001 yg | 0.07M | NS | 0.001 |  | <0.001 |  |  |  |  |  |
|  |  |  |  | MV AC | 0.001 yg | NS |  | 0.001 |  | 0.08 |  |  |  |  |  |
| Rodrig 2018[35] | 182/145, ret | 1d-1y | NG OA | UV AC |  |  |  |  | NS | NS |  |  |  |  |  |
| DeVience 2017[15] | 115/115, ret | 3y | NG | UV %C | NS | NS | NS | r=0.35 | NS | NS | NS |  | 0.03 | NS | phacotime 0.02 |
|  |  |  |  | MV %C | NS |  |  | 0.01 |  |  |  |  | NS |  | phacotime 0.038 |
| Lee 2016[16] | 161/116, pros | 1d, 1m, 3m | NG | UV AC |  |  |  |  |  |  |  |  |  |  | CDE NS |
|  |  |  |  | MV AC |  |  |  |  |  |  |  |  |  |  | CDE NS |
| Sengupta 2016[17] | 500/500 MSICS/PE, RCT | 6m | NG | MV AC | NS | NS |  | <0.001 |  | NS |  |  |  |  | MSICS NS |
| Coh 2016[41] | 97/73, pros | 4m | NG | UV %C & AC | NS | 0.004M |  | <0.001 | <0.001 | <0.001 | 0.10 | <0.001 | NS |  |  |
|  |  |  |  | MV %C & AC |  |  |  |  | <0.001 | <0.001 | 0.07 | <0.001 | NS |  |  |
| Anazi 2016[42] | 252/211, ret | 1y | NG | UV AC | NS | NS | NS | 0.03 |  | NS |  |  |  |  |  |
| Ngo 2016[18] | 116/116, ret | 1, 3, 6, 12,24m | NG no PACS | UV AC |  |  |  | 0.01 r=0.48–0.56 |  |  |  |  |  |  | ECCE NS |
| Park 2016 Korea[83] | 29/29, pros | 1m | NG | UV AC |  |  |  | <0.01 r=0.52 | <0.05 r=0.38 | NS |  |  |  |  |  |
| Moghimi 2015[43] | 85/85, pros | 3m | NG OA | UV AC | NS | NS |  | 0.001 | 0.03 | <0.001 | NS |  |  |  |  |
|  |  |  |  | MV AC | NS | NS |  | 0.03 | NS | 0.002 |  |  |  |  |  |
| Zetterstrom 2015[44] | 20437/?, ret | 38d | (NG) | UV AC | 0.001 old | 0.019F |  | r=0.56 |  |  |  |  |  |  |  |
|  |  |  |  | MV AC | 0.001 old | NS |  | 0.001 |  | 0.001 |  |  |  |  |  |
| Hsu 2015[45] | 75/75, pros | 4m | NG OA | UV %C | NS | 0.06M |  | r=0.43 | <0.001 | <0.001 | 0.039 |  |  |  |  |
|  |  |  |  | MV AC & %C | NS | NS |  | 0.006 | 0.003 | 0.052 | 0.020 | <0.001 | NS |  |  |
|  |  |  |  | UV AC |  |  |  | r=0.55 |  |  |  |  |  |  |  |
| Bilak 2015[46] | 117/117, pros | 1m | NG | UV AC |  |  |  | 0.01 | NS | NS |  |  |  |  |  |
|  |  |  |  | MV AC |  |  |  | 0.000 |  |  |  |  |  |  |  |
| Yang 2013[47] | 999/999, ret | 3m | NG | UV AC |  |  |  | r=0.40 | 0.013 | NS | <0.001 |  |  |  |  |
|  |  |  |  | MV AC |  |  |  | 0.0001 | 0.07 |  | <0.001 |  |  |  |  |
| Huang 2012[48] | 73/73, pros | 3m | NG | UV AC |  |  |  | r=0.25 |  |  |  |  |  |  |  |
| Pradhan 2012[49] | 77/77, ret | 2-18m | 13% GC, all OA | UV A^iop^ | NS |  |  | NS | NS |  | NS |  |  |  | phacotime NS |
|  |  |  |  | MV A^iop^ |  |  |  | 0.001 |  |  |  |  |  |  |  |
| Mansberger 2012[50] | 63/42, post RCT | 1y | OHT NG OA | UV A^iop^ | NS | NS |  | r=0.53 |  |  |  |  |  |  |  |
|  |  |  |  | MV A^iop^ | NS | NS |  | 0.001 |  |  |  |  |  |  |  |
| Huang 2011[51] | 63/49, pros | 6m | NG, 37 OA,  26 NA | UV AC |  |  |  |  | 0.08 |  |  |  |  |  |  |
|  |  |  |  | MV AC |  |  |  |  |  | NS |  |  |  |  |  |
| Dooley 2010[52] | 101/101, pros | 6w | NG | UV AC | NS | 0.01M |  |  | NS |  |  |  |  |  |  |
| Irak 2010[20] | 266/266, ret | 2y | NG | UV AC |  |  |  |  | NS | NS | 0.02 |  |  |  |  |
| Shin 2010[53] | 70/70, pros | 3m | NG  35 OA  35 NA | MV AC | NS | NS |  | r=0.565 OA r=0.708 NA p<0.01 | NS | NS | NS |  |  |  |  |
| Poley 2008[54] | 588/?, ret | 1y | NG | UV AC | NS |  |  | 0.001 |  |  |  |  |  |  |  |
| Issa 2005[55] | 103/103, pros | 2m | NG | UV AC |  |  | NS | r=0.75 |  |  |  |  |  |  |  |
|  |  |  |  | MV AC | NS | NS |  |  | <0.01 | NS | NS |  |  |  |  |
| Altan 2004[23] | 53/49, pros | 6m | NG OA | UV AC |  |  |  |  | NS |  |  |  |  |  |  |
| Pohjalainen 2001[24] | 137/?, ret | 1d, 4m, 1-2.7y | NG | UV AC | All NS |  |  |  |  |  |  |  |  |  | phacotime NS |
|  |  | 1w |  |  | NS |  |  |  |  |  |  |  |  |  | phacotime 0.01 |
| Kee 2000[91] | 42/42, pros | 2m | NG |  |  |  |  |  |  |  |  |  |  |  |  |
| Tong 1998[28] | 385/303, ret | 6-8m | NG | UV AC | NS | NS |  |  |  |  |  |  |  |  |  |
| Suzuki 1997[56] | 498/498, ret | 6y | NG | UV AC |  |  |  | assoc |  |  |  |  |  |  |  |

? not clear whether bilateral eyes were sometimes included.

%C, percentage change in IOP; AC, absolute change in IOP; ACD, anterior chamber depth; A^iop^, absolute IOP value; assoc, associated; AXL, axial length; CDE, cumulative dissipated energy; d, day; ECCE, extracapsular cataract extraction; F, female; FLACS, femtosecond laser-assisted cataract surgery; EPT, estimated phaco time; GC, glaucomatous; IOP, intraocular pressure; m, months; LP, lens position; M, male; MSICS, manual small-incision cataract surgery; MV, multivariable; NA, narrow angle; NG, nonglaucomatous; (NG), probably mostly nonglaucomatous; NS, not significant; OA, open angle; old, older; PACS, primary angle-closure suspect; PE, phacoemulsification; pros, prospective; ret, retrospective; RCT, randomized clinical trial; UV, univariable; w, weeks; y, years; yg, younger.
