# Supplementary material for: Evolution of intraocular pressure after cataract surgery in nonglaucomatous patients: A post-hoc analysis of PERCEPOLIS clinical trial data
Source: PLoS One. 2026 May 19;21(5):e0349310. doi: 10.1371/journal.pone.0349310 (PMC13186369; doi:10.1371/journal.pone.0349310)
Supplement: S3 Table — (DOCX) [file pone.0349310.s007.docx]

### S3 Table. Previous studies on ocular macroanatomical measurements that associated with or predicted IOP drop after cataract surgery in nonglaucomatous or glaucomatous eyes

| **Association with ↓preACD** | **No association with preACD** | **Association with ↑preACD** |
| --- | --- | --- |
| ***Nonglaucomatous*** | | |
| Issa 2005[55] NG UV | Altan 2004[23] NG UV |  |
| Yang 2013[47] NG UV →→→ | Yang 2013[47] NG MV |  |
| Moghimi 2015[43] NG UV→→ | Moghimi 2015[43] NG MV |  |
| Coh 2016[41] NG UV+MV | Shin 2010[53] NG MV |  |
| Uzun 2025[58] NG UV+MV | Dooley 2010[52] NG UV |  |
|  | Ramli 2019[32] NG UV |  |
|  | Ramez 2021[39] NG UV |  |
|  | Bilak 2015[46] NG UV |  |
| ***Nonglaucomatous-open angle*** | | |
| Hsu 2015[45] NG-OA UV+MV | Pradhan 2012[49] NG-OA UV |  |
|  | Rodrigues 2018[35] NG-OA UV |  |
|  | Markic 2022[29] NG-OA UV+MV |  |
| ***Glaucomatous*** | | |
| Perez 2019[65] GC UV+MV | Yoo 2018[66] GS UV+MV |  |
| Liu 2006[67] PACG MV | Eslami 2013[69] PACG UV |  |
| Liu 2011[68] PACG MV | Latifi 2013[70] PACG UV |  |
|  | Coh 2016[41] POAG UV+MV | Slabaugh 2014[74] POAG MV |
|  | Hsia 2017[71] POAG UV |  |
|  | Lin 2017[72] POAG MV |  |
|  | Yoo 2018[66] POAG UV+MV |  |
|  | Ramez 2021[39] PXS UV |  |
|  | Moghimi 2017[73] PXS UV |  |
|  | Markic 2022[29] PXG UV+MV |  |
| **Deepening of ACD** | **No association with ACD change** |  |
| Huang 2011[51] NG UV p=0.08 | Bilak 2015[46] NG UV |  |
| Huang 2012[48] NG UV | Sengupta 2016[17] NG MV |  |
|  | Xirou 2023[37] NG UV |  |
|  | Xirou 2023[37] POAG UV |  |
|  | Dawood 2023[57] NG UV |  |
| **Assoc with ↓preAXL** | **No association with preAXL** | **Association with ↑preAXL** |
| Zetterstrom 2015[44] NG UV | Yang 2013[47] NG UV |  |
| Moghimi 2015[43] NG UV+MV | Sengupta 2016[17] NG MV |  |
| Ramli 2019[32] NG MV | Devience 2017[15] NG UV |  |
| Zetterstrom 2015[44] NG UV | Bilak 2015[46] NG UV |  |
|  | Anazi 2016[42] NG UV |  |
|  | Baek 2019[34] (NG) MV ←←←← | Baek 2019[34] (NG) UV |
|  | Beato 2019[33] NG-OA UV + MV |  |
|  | Markic 2022[29] NG-OA UV+MV | Markic 2022[29] PXG UV+MV |
|  | Uzun 2025[58] NG UV+MV |  |
| Coh 2016[41] NG UV+MV | Coh 2016[41] POAG UV+MV |  |
|  | Moghimi 2017[73] PXS UV |  |
| Hsu 2015[45] NG-OA UV →→ | Hsu 2015[45] NG-OA MV |  |
| Yoo 2018[66] GS UV+MV | Dawood 2023[57] NG UV |  |
| Perez 2019[65] GC UV →→→ | Perez 2019[65] GC MV |  |
|  | Hsia 2017[71] POAG UV |  |
|  | Yoo 2018[66] POAG UV+MV |  |
| **Association with ↑LT** | **No association with LT** |  |
| Yang 2013[47] NG UV+MV | Issa 2005[55] NG UV |  |
| Bilak 2015[46] NG UV | Shin 2010[53] NG MV |  |
| Hsu 2015[45] NG-OA UV+MV | Coh 2016[41] NG UV |  |
| Uzun 2025[58] NG UV+MV | Ramli 2019[32] NG UV+MV |  |
| Perez 2019[65] GC UV+MV | Devience 2017[15] NG UV |  |
|  | Moghimi 2015[43] NG UV |  |
|  | Pradhan 2012[49] NG-OA UV |  |
|  | Markic 2022[29] NG-OA UV+MV |  |
|  | Markic 2022[29] PXG UV+MV |  |
|  | Yoo 2018[66] GS UV+MV |  |
|  | Yoo 2018[66] POAG UV+MV |  |
| Kader 2022[14] PACS/PACG UV | Kader 2022[14] NG & POAG UV |  |
|  | Liu 2006[67] PACG MV |  |
|  | Liu 2011[68] PACG MV |  |
|  | Yudha 2012[75] PACG UV |  |
| **Association with ↑prePD ratio** | **No association with prePD ratio** |  |
| Issa 2005[55] NG UV |  |  |
| Dooley 2010[52] NG UV |  |  |
| Coh 2016[41] NG UV+MV |  |  |
| Hsu 2015[45] NG-OA UV+MV |  |  |
| Beato 2019[33] NG-OA UV →→ | Beato 2019[33] NG-OA MV |  |
| Markic 2022[29] NG-OA UV+MV |  |  |
| Markic 2022[29] PXG UV+MV |  |  |
| Yoo 2018[66] GS UV+MV |  |  |
| Yoo 2018[66] POAG UV+MV |  |  |
| **Association with anterior (↓) preLP** | **No association with preLP** |  |
| Hsu 2015[45] NG-OA UV+MV | Ramli 2019[32] NG UV |  |
| Coh 2016[41] NG UV+MV | Markic 2022[29] NG-OA UV+MV |  |
|  | Markic 2022[29] PXG UV+MV |  |
|  | Yoo 2018[66] POAG UV+MV |  |
|  | Yoo 2018[66] GS UV+MV |  |
| **Association with anterior (↓) preRLP** | **No association with preRLP** |  |
|  | Hsu 2015[45] NG-OA MV |  |
|  | Coh 2016[41] NG UV |  |
| Devience 2017[15] NG UV→→ | Devience 2017[15] NG MV |  |
|  | Ramli 2019[32] NG UV |  |
| Markic 2022[29] PXG MV | Markic 2022[29] NG-OA UV+MV |  |
| **Association with ↓preACA** | **No association with preACA (widening)[postACD]** | **Association with ACA widening** |
| Moghimi 2015[43] NG UV | (Altan 2004[23] NG UV) |  |
| Yang 2013[47] NG UV | Dooley 2010[52] NG UV | Yang 2013[47] NG UV+MV |
| Shams 2012[76] PAC UV | Perez 2019[65] NG UV |  |
| Mayer 2015[77] GC UV+MV | Ramli 2019[32] NG UV |  |
|  | Ramez 2021[39] NG UV |  |
|  | Pradhan 2012[49] NG-OA UV |  |
|  | [Pradhan 2012[49] NG-OA UV] |  |
|  | Lin 2017[72] POAG MV |  |
|  | Moghimi 2017[73] PXS UV |  |
|  | Ramez 2021[39] PXS UV |  |
|  | (Dawood 2023[57] NG UV) |  |
| **Association with ↓ACW** | **No association with ACW [ACW change]** |  |
| Moghimi 2015[43] NG-OA UV→ | Moghimi 2015[43] NG-OA MV |  |
|  | [Moghimi 2017[73] PXS UV] |  |
|  | Hsia 2017[71] POAG UV |  |
|  | Lin 2017[72] POAG MV |  |
|  | Perez 2019[65] NG UV |  |
|  | [Xirou 2023[37] NG UV] |  |
|  | [Xirou 2023[37] POAG UV] |  |
| **Association with ↓preAOD** | **No association with preAOD [AOD change]** | **Association with AOD widening** |
| Yang 2013[47] NG AOD^500^ UV →→ | Yang 2013[47] NG AOD^500^ MV | Yang 2013[47] NG AOD^500^ UV+MV |
| Moghimi 2015[43] NG AOD^500^ UV → | Moghimi 2015[43] NG AOD^500^ MV | Huang 2011[51] NG AOD^500^ MV |
|  | [Sengupta 2016[17] NG AOD^500^ MV] | Huang 2012[48] NG AOD^500^ UV |
|  | Moghimi 2017[73] PXS AOD^500^ AOD^750^ UV |  |
|  | Ramli 2019[32] NG AOD^750^ UV |  |
|  | Ramez 2021[39] NG AOD^500^ UV |  |
|  | [Ramez 2021[39] NG AOD^500^ UV] |  |
|  | Xirou 2023[37] NG AOD^500^ UV |  |
| Hsia 2017[71] POAG AOD^500^ AOD^750^ UV+MV | Xirou 2023[37] POAG AOD^500^ UV |  |
| Lin 2017[72] POAG AOD^500^ UV+MV | Latifi 2013[70] PACG AOD^500^ AOD^750^ UV |  |
| Perez 2019[65] GC AOD^750^ UV+MV | Eslami 2013[69] PACG AOD^500^ AOD^750^ UV |  |
|  | Perez 2019[65] GC AOD^50^ UV |  |
|  | Ramez 2021[39] PXS AOD^500^ UV |  |
|  | [Ramez 2021[39] PXS AOD^500^ UV] |  |
|  | [Dawood 2023[57] NG UV] |  |
| **Association with ↓preTISA** | **No association with preTISA [TISA change]** | **Association with TISA widening** |
| Moghimi 2015[43] NG TISA^500^ UV | Eslami 2013[69] PACG TISA^500^ TISA^750^ UV |  |
|  | Latifi 2013[70] PACG TISA^500^ AOD^750^ UV |  |
| Hsia 2017[71] POAG TISA^750^ UV → | Hsia 2017[71] POAG TISA^750^ MV |  |
|  | Hsia 2017[71] POAG TISA^500^ UV |  |
|  | Perez 2019[65] GC TISA^750^ UV+MV |  |
|  | Ramez 2021[39] NG TISA^500^ UV |  |
|  | [Ramez 2021[39] NG TISA^500^ UV] |  |
|  | Ramez 2021[39] PXS TISA^500^ UV |  |
|  | [Ramez 2021[39] PXS TISA^500^ UV] |  |
| **Association with ↑preLV** | **No association with preLV** | **Association with LV increase** |
| Hsia 2017[71] POAG UV+MV | Eslami 2013[69] PACG UV | Huang 2012[48] NG UV |
| Lin 2017[72] POAG MV | Latifi 2013[70] PACG UV |  |
| Perez 2019[65] GC UV+MV | Moghimi 2015[43] NG UV |  |
|  | Moghimi 2017[73] PXS UV |  |
| Perez 2019[65] GC UV →→→→ | Perez 2019[65] GC MV |  |
|  | Ramli 2019[32] NG UV |  |
| **Association with ↑preRLV** | **No association with preRLV** | **Association with RLV increase** |
|  | Ramli 2019[32] NG UV |  |
|  | Moghimi 2015[43] NG UV |  |
| **Association with ↓preAV** | **No association with preAV** | **Association with AV decrease** |
| Moghimi 2015[43] NG UV+MV | Hsia 2017[71] POAG UV |  |
|  | Ramli 2019[32] NG UV |  |
| **Association with ↑preIcurv** | **No association with preIcurv** | **Association with Icurv decrease** |
|  | Huang 2012[48] NG UV |  |
|  | Moghimi 2015[43] NG UV |  |
|  | Moghimi 2017[73] PXS UV |  |
|  | Hsia 2017[71] POAG UV |  |
|  | Lin 2017[72] POAG MV |  |
|  | Perez 2019[65] GC UV |  |
|  | Ramli 2019[32] NG UV |  |
| **Association with ↑preIarea** | **No association with preIarea** | **Association with Iarea decrease** |
| Pradhan 2012[49] NG-OA UV+MV | Moghimi 2015[43] NG UV |  |
| Lin 2017[72] POAG MV | Moghimi 2017[73] PXS UV |  |
|  | Hsia 2017[71] POAG UV |  |
|  | Perez 2019[65] GC UV |  |
|  | Ramli 2019[32] NG UV |  |
| **Association with ↑preIT** | **No association with preIT** | **Association with IT decrease** |
| Lin 2017[72] IT^750 2000^ POAG MV | Yang 2013[47] NG IT^750^ MV |  |
|  | Moghimi 2015[43] IT^750^ IT^2000^ NG UV |  |
|  | Moghimi 2017[73] IT^750^ PXS UV |  |
|  | Hsia 2017[71] IT^750^ IT^2000^ POAG UV |  |
|  | Perez 2019[65] IT^750^ IT^2000^ GC UV |  |
|  | Ramli 2019[32] IT^750^ NG UV |  |

ACA, anterior chamber angle; ACD, anterior chamber depth; ACW, anterior chamber width; AOD, angle-opening distance (the angle-opening distance 500 or 750 μm from the scleral spur; AXL, axial length; GC, unspecified glaucoma; Iarea, area of the iris; Icurv, iris curvature (a line is drawn from the most peripheral to the most central point of the iris pigmented epithelium, and a perpendicular line is extended from this line to the iris pigmented epithelium at the point of greatest convexity; Icurv=length of latter line); IT, iris thickness at 750 or 2000 µm from the scleral spur; LP, lens position (calculated as ACD+0.5LT); LV, lens vault (the perpendicular distance between the anterior crystalline lens surface and the horizontal line joining the two scleral spurs); MV, multivariable analysis; OA, open angle; PCAG, primary closed angle glaucoma; POAG, primary open angle glaucoma; pre, preoperative; PXG, pseudoexfoliation glaucoma; PXS, pseudoexfoliation syndrome without glaucoma; RLP, relative lens position (calculated as LP/AXL); TISA, the trapezoidal area at the scleral spur (the boundaries are: anteriorly, a perpendicular line between the inner corneoscleral wall and the iris surface at 500 or 750 μm anterior to the scleral spur; posteriorly, a line perpendicular to the inner corneoscleral wall extending from the scleral spur to the iris surface; superiorly, the inner corneoscleral wall; and inferiorly, the iris surface); UV, univariable analysis.
