# Supplementary material for: Evolution of intraocular pressure after cataract surgery in nonglaucomatous patients: A post-hoc analysis of PERCEPOLIS clinical trial data
Source: PLoS One. 2026 May 19;21(5):e0349310. doi: 10.1371/journal.pone.0349310 (PMC13186369; doi:10.1371/journal.pone.0349310)
Supplement: S4 Table — (DOCX) [file pone.0349310.s008.docx]

### S4 Table. Univariable comparison of the 3-month whole cohort (*n*=241) and the 12-month (*n*=173) patients in terms of demographic, preoperative clinical, and operative characteristics

| Variable | M3 patients (*n*=241)  Mean ± SD or *n* (%) | M12 patients (*n*=173)  Mean ± SD or *n* (%) | P* |
| --- | --- | --- | --- |
| Age, years | 74 ± 9 | 73 ± 10 | 0.36 |
| Female sex | 136 (56) | 101 (58) | 0.92 |
| Cataract density |  |  | 0.86 |
| N1/2 | 46 (19) | 34 (20) |  |
| N3 | 116 (48) | 87 (50) |  |
| N4/5 | 79 (33) | 52 (30) |  |
| Preoperative IOP, mmHg | 17.6 ± 3.5 | 17.6 ± 3.7 | 0.96 |
| Preoperative ACD, mm | 3.1 ± 0.4 | 3.1 ± 0.4 | 0.68 |
| Preoperative AXL, mm | 23.2 ± 0.8 | 23.3 ± 0.7 | 0.73 |
| Preoperative LT, mm | 4.6 ± 0.4 | 4.6 ± 0.4 | 0.75 |
| Surgical technique |  |  | 0.92 |
| Subluxation | 125 (52) | 97 (56) |  |
| DAC | 116 (48) | 76 (44) |  |
| EPT, seconds | 6 ± 3 | 6 ± 3 | 0.31 |
| Implant power, D | 21 ± 4 | 20 ± 5 | 0.30 |

*P values were determined by Student’s *t-*test or Chi-squared test.

DAC, divide-and-conquer; EPT, effective phaco time; IOP, intraocular pressure.
