# Supplementary material for: Evolution of intraocular pressure after cataract surgery in nonglaucomatous patients: A post-hoc analysis of PERCEPOLIS clinical trial data
Source: PLoS One. 2026 May 19;21(5):e0349310. doi: 10.1371/journal.pone.0349310 (PMC13186369; doi:10.1371/journal.pone.0349310)
Supplement: S5 Table — (DOCX) [file pone.0349310.s009.docx]

### S5 Table. Preliminary multiple linear regression analysis of the ability of pre/perioperative variables to predict absolute IOP change (mmHg) and % IOP change at 3 months (*n*=241)

|  | % change in IOP | | | | Absolute change in IOP, mmHg | | | |
| --- | --- | --- | --- | --- | --- | --- | --- | --- |
| Variable | | Beta ± sd | Partial R² | *p* | | Beta ± sd | Partial R² | *p* |
| Age, years | | -0.01 ± 0.01 | 0.01 | 0.14 | | 0.04 ± 0.02 | 0.01 | **0.04** |
| Sex | | -0.05 ± 0.02 | 0.02 | **0.01** | | -0.80 ± 0.31 | 0.02 | **0.01** |
| Cataract density  N1/2  N3  N4/5 | | -0.01 ± 0.03  Ref.  0.02 ± 0.02 | 0.003 | 0.88  Ref.  0.37 | | 0.32 ± 0.43  Ref.  0.16 ± 0.34 | 0.003 | 0.45  Ref.  0.65 |
| Preop IOP, mmHg | | -0.02 ± 0.01 | 0.21 | **<0.001** | | 0.53 ± 0.04 | 0.37 | **<0.001** |
| Subluxation surgery | | -0.01 ± 0.02 | 0.001 | 0.53 | | -0.20 ± 0.30 | 0.001 | 0.50 |
| EPT, seconds | | 0.01 ± 0.01 | 0.002 | 0.49 | | 0.04 ±0.05 | 0.001 | 0.50 |
| Implant power, D | | -0.01 ± 0.01 | <0.001 | 0.82 | | 0.01 ± 0.04 | <0.001 | 0.90 |

EPT, effective phaco time; IOP, intraocular pressure; preop, preoperative; Ref., reference.
