# Supplementary material for: Evolution of intraocular pressure after cataract surgery in nonglaucomatous patients: A post-hoc analysis of PERCEPOLIS clinical trial data
Source: PLoS One. 2026 May 19;21(5):e0349310. doi: 10.1371/journal.pone.0349310 (PMC13186369; doi:10.1371/journal.pone.0349310)
Supplement: S6 Table — (DOCX) [file pone.0349310.s010.docx]

### S6 Table. Preliminary multivariable analysis of the ability of pre/perioperative variables to predict IOP change (mmHg) in the 3-month cohort only and both the 3- and 12-month cohorts (*n*=241)

| Variable | Absolute change in IOP, mmHg | | | | | |
| --- | --- | --- | --- | --- | --- | --- |
|  | **3-month cohort only^a^** | | | **3- and 12-month cohorts^b^** | | |
|  | **Beta ± SD** | **Partial r²** | *p* | **Beta ± SD** | **Partial r²** | ***p*** |
| Time (3 *vs*. 12 months) | - | - |  | -0.72**±**0.25 | 0.01 | **0.003** |
| Age, years | 0.04 ± 0.02 | 0.01 | **0.04** | 0.0002**±**0.02 | <0.001 | 0.99 |
| Female sex | -0.80 ± 0.31 | 0.02 | **0.01** | -0.64±0.25 | 0.01 | **0.01** |
| Cataract density  N1/2  N3  N4/5 | Ref.  0.32 ± 0.43  0.48 ± 0.46 | 0.003 | Ref.  0.45  0.29 | Ref.  0.42±0.34  0.35±0.37 | 0.002 | Ref.  0352  0.54 |
| Preoperative IOP, mmHg | 0.53 ± 0.04 | 0.37 | **<0.001** | -0.55±0.04 | 0.36 | **<0.0001** |
| Subluxation surgery | -0.20 ± 0.30 | 0.001 | 0.50 | -0.25±0.25 | 0.002 | 0.31 |
| EPT, seconds | 0.04 ±0.05 | 0.001 | 0.50 | 0.01±0.04 | <0.001 | 0.87 |
| Implant power, D | 0.01 ± 0.04 | <0.001 | 0.90 | -0.03±0.03 | 0.001 | 0.36 |

^a^ Multiple linear regression analysis.

^b^ Linear mixed-effects model.

EPT, effective phaco time; IOP, intraocular pressure; Ref., reference; SD, standard deviation.
