# Supplementary material for: Evolution of intraocular pressure after cataract surgery in nonglaucomatous patients: A post-hoc analysis of PERCEPOLIS clinical trial data
Source: PLoS One. 2026 May 19;21(5):e0349310. doi: 10.1371/journal.pone.0349310 (PMC13186369; doi:10.1371/journal.pone.0349310)
Supplement: S7 Table — (DOCX) [file pone.0349310.s011.docx]

### S7 Table. Univariable analysis of the association between absolute IOP change (mmHg) at 3 months and pre/perioperative variables in the 3-month whole cohort (*n*=241)

| Characteristic | *n* | r [95%CI] or mean ± SD | *p** |
| --- | --- | --- | --- |
| Age | 241 | r=-0.03 [-0.16; 0.09] | 0.62 |
| Sex |  |  | 0.25 |
| Female | 136 | 2.2 ± 2.9 |  |
| Male | 105 | 2.6 ± 3.0 |  |
| Cataract density |  |  | 0.64 |
| N1/2 | 46 | 2.3 ± 3.3 |  |
| N3 | 116 | 2.6 ± 2.5 |  |
| N4/5 | 79 | 2.2 ± 3.3 |  |
| Preoperative IOP | 241 | r=0.60 [-0.67; -0.51] | **<0.0001** |
| Preoperative ACD | 240 | r=-0.05 [-0.17; 0.08] | 0.47 |
| Preoperative AXL | 240 | r=0.03 [-0.09; 0.16] | 0.62 |
| Preoperative LT | 240 | r=0.09 [-0.04; 0.21] | 0.17 |
| Preoperative LP | 240 | r=-0.01 [-0.13; 0.12] | 0.92 |
| Preoperative RLP | 240 | r=-0.03 [-0.15; 0.10] | 0.69 |
| Surgical technique |  |  | 0.22 |
| Subluxation | 125 | 2.6 ± 3.0 |  |
| DAC | 116 | 2.2 ± 2.9 |  |
| EPT | 241 | r=0.04 [-0.09; 0.16] | 0.57 |
| Implant power |  |  | 0.83 |
| <21 D | 105 | 2.4 ± 2.8 |  |
| ≥21 D | 136 | 2.4 ± 3.0 |  |

*Student’s *t-*test, ANOVA, or Pearson correlation analysis.

ACD, anterior chamber depth; AXL, axial length; CI, confidence intervals; D, diopters; DAC, divide-and-conquer; EPT, effective phaco time; IOP, intraocular pressure; LP, lens position (ACD+0.5LT); LT, lens thickness; RLP, relative lens position (LP/AXL); SD, standard deviation.
