# Supplementary material for: Evolution of intraocular pressure after cataract surgery in nonglaucomatous patients: A post-hoc analysis of PERCEPOLIS clinical trial data
Source: PLoS One. 2026 May 19;21(5):e0349310. doi: 10.1371/journal.pone.0349310 (PMC13186369; doi:10.1371/journal.pone.0349310)
Supplement: S8 Table — (DOCX) [file pone.0349310.s012.docx]

### S8 Table. Multiple linear regression analysis of the ability of LP and RLP to predict absolute IOP change at 3 months in the whole cohort (*n*=241)

|  | Beta ± SD | Partial r² | *p* | Beta ± SD | Partial r² | *p* |
| --- | --- | --- | --- | --- | --- | --- |
| Age, years | 0.04 ± 0.02 | 0.01 | 0.055 | 0.04 ± 0.02 | 0.01 | 0.056 |
| Female sex | 0.79 ± 0.31 | 0.02 | **0.01** | 0.78 ± 0.31 | 0.02 | **0.01** |
| Cataract density  N1/2  N3  N4/5 | Ref.  -0.25 ± 0.43  -0.39 ± 0.46 | 0.002 | Ref.  0.55  0.40 | Ref.  -0.26 ± 0.43  -0.38 ± 0.46 | 0.002 | Ref.  0.54  0.41 |
| Preoperative IOP, mm | 0.54 ± 0.04 | 0.37 | **<0.001** | 0.53 ± 0.04 | 0.37 | **<0.001** |
| Preoperative AXL, mm | -0.03 ± 0.24 | <0.001 | 0.89 | Excluded due to collinearity with RLP | | |
| Preoperative LP | 0.63 ± 0.46 | 0.005 | 0.17 | Excluded due to collinearity with RLP | | |
| Preoperative RLP | Excluded due to collinearity with AXL | | | 15.04 ± 10.68 | 0.005 | 0.16 |
| Subluxation surgery | 0.2 ± 0.31 | 0.2 ± 0.31 | 0.001 | 0.52 |  |  |
| EPT, seconds | -0.04 ±0.05 | -0.04 ±0.05 | 0.002 | 0.43 |  |  |
| Implant power, D | 0.02 ± 0.04 | 0.02 ± 0.04 | <0.001 | 0.70 |  |  |

ACD, anterior chamber depth; AXL, axial length; EPT, effective phaco time; IOP, intraocular pressure; LP, lens position (ACD+0.5LT); LT, lens thickness; Ref., reference; RLP, relative-lens position (LP/AXL); SD, standard deviation.
