# Supplementary material for: Evolution of intraocular pressure after cataract surgery in nonglaucomatous patients: A post-hoc analysis of PERCEPOLIS clinical trial data
Source: PLoS One. 2026 May 19;21(5):e0349310. doi: 10.1371/journal.pone.0349310 (PMC13186369; doi:10.1371/journal.pone.0349310)
Supplement: S9 Table — (DOCX) [file pone.0349310.s013.docx]

### S9 Table. Multiple linear regression analysis of the ability of pre/perioperative variables, including RLP, to predict absolute IOP change at 3 months in eye subgroups with preoperative IOP of 20–29, 15–19, or 10–14 mmHg (total *n*=241)

| Variable | Preoperative IOP 20–29 mmHg  (High subgroup) *n*=75 | | | Preoperative IOP 15–19 mmHg  (Intermediate subgroup) *n*=117 | | | | Preoperative IOP 10–14 mmHg  (Low subgroup) *n*=49 | | | |
| --- | --- | --- | --- | --- | --- | --- | --- | --- | --- | --- | --- |
|  | Beta ± SD | Partial r² | *p* | | Beta ± SD | Partial r² | *p* | | Beta ± SD | Partial r² | *p* |
| Age, years | 0.06 ± 0.03 | 0.02 | 0.10 | | 0.01 ± 0.03 | 0.001 | 0.69 | | 0.02 ± 0.05 | 0.003 | 0.66 |
| Female sex | 1.59 ± 0.56 | 0.07 | **0.01** | | 0.49 ± 0.46 | 0.01 | 0.28 | | 0.39 ± 0.67 | 0.006 | 0.56 |
| Cataract density  N1/2  N3  N4/5 | Ref.  -0.73 ± 0.69  -0.2 ± 0.79 | 0.01 | Ref.  0.30  0.80 | | Ref.  -0.83 ± 0.72  -0.91 ± 0.72 | 0.01 | Ref.  0.25  0.21 | | Ref.  1.06 ± 0.76  -1.03 ± 0.99 | 0.11 | Ref.  0,17  0,31 |
| Preop IOP, mmHg | 0.79 ± 0.14 | 0.26 | **<0.0001** | | 0.34 ± 0.17 | 0.03 | **0.049** | | 0.64 ± 0.29 | 0.08 | **0.03** |
| Preop RPL | 7.48 ± 17.96 | 0.001 | 0.68 | | 15.2 ± 16.41 | 0.01 | 0.36 | | 19.29 ± 22.17 | 0.01 | 0.39 |
| Subluxation surg | 0.06 ± 0.53 | <0.001 | 0.92 | | -0.03 ± 0.46 | <0.001 | 0.95 | | 0.92 ± 0.68 | 0.03 | 0.18 |
| EPT, seconds | -0.09 ± 0.09 | 0.01 | 0.31 | | -0.07 ± 0.08 | 0.01 | 0.39 | | 0.14 ± 0.12 | 0.02 | 0.25 |
| Implant power, D | -0.1 ± 0.06 | 0.02 | 0.11 | | 0.19 ± 0.07 | 0.06 | **0.01** | | -0.03 ± 0.09 | 0.001 | 0.77 |

ACD, anterior chamber depth; AXL, axial length; EPT, effective phaco time; IOP, intraocular pressure; LT, lens thickness; preop, preoperative; RLP, relative lens position ([ACD+0.5LT]/AXL); SD, standard deviation; surg, surgery.
