# Supplementary material for: Evolution of intraocular pressure after cataract surgery in nonglaucomatous patients: A post-hoc analysis of PERCEPOLIS clinical trial data
Source: PLoS One. 2026 May 19;21(5):e0349310. doi: 10.1371/journal.pone.0349310 (PMC13186369; doi:10.1371/journal.pone.0349310)
Supplement: S10 Table — (DOCX) [file pone.0349310.s014.docx]

### S10 Table. Summary of studies that controlled for regression-to-the-mean when assessing the effect of cataract surgery on IOP

| **Study year country^Ref^** | **Study design (study period)** | **Type of eye** | **No. preop IOP measurements** | **No. eyes/pt** | **Age, y** | **Preop IOP, mmHg** | **Follow-up** | **Absolute IOP change, mmHg** | **% IOP change** |
| --- | --- | --- | --- | --- | --- | --- | --- | --- | --- |
| Mansberger 2012 USA[50] | Post-hoc OHTS RCT: effect of IOP meds on POAG onset (1994–1996) | High IOP (>21 mmHg), no glaucomatous damage, naïve for IOP-lowering meds, open angle: cataract surgery | 3 (RTM control) | 63/42 | 64 | Mean=23.9 | 1 w | -4.8 | -20% |
|  |  |  |  |  |  |  | 6 m | -3.9 | -16% |
|  |  |  |  |  |  |  | 12 m | -3.6 | -15% |
|  |  |  |  |  |  |  | 18 m | -3.4 | -14% |
|  |  |  |  |  |  |  | 24 m | -3.3 | -14% |
|  |  |  |  |  |  |  | 30 m | -3.7 | -16% |
|  |  |  |  |  |  |  | 36 m | -3.3 | -14% |
|  |  | IOP-matched control group with high IOP (>21 mmHg), no glaucomatous damage, naïve for IOP-lowering meds, open angle: no cataract surgery (RTM control) | 3 (RTM control) | 743/743 | 55 | Mean=23.8 | 1 w | 0 | 0% |
|  |  |  |  |  |  |  | 6 m | 0 | 0% |
|  |  |  |  |  |  |  | 12 m | -0.5 | -2% |
|  |  |  |  |  |  |  | 18 m | -0.6 | -3% |
|  |  |  |  |  |  |  | 24 m | -0.7 | -3% |
|  |  |  |  |  |  |  | 30 m | -0.8 | -3% |
|  |  |  |  |  |  |  | 36 m | -0.8 | -3% |
| Qassim 2020 Australia[6] | Pros (period?) | Probable early POAG: cataract surgery | 7 (RTM control) | 171/108 | 69 | Mean=16.7 | 6 m | -2.2 | -13% |
|  |  |  |  |  |  |  | 12 m | -2.4 | -15% |
|  |  |  |  |  |  |  | 18 m | -2.0 | -12% |
|  |  |  |  |  |  |  | 24 m | -1.7 | -10% |
|  |  |  |  |  |  |  | 30 m | -1.9 | -11% |
|  |  |  |  |  |  |  | 36 m | -1.6 | -10% |
|  |  |  |  |  |  |  | 42 m | -1.8 | -11% |
|  |  |  |  |  |  |  | 48 m | -1.4 | -8% |
|  |  | IOP-, age-, and sex-matched control group with Probable early POAG: no cataract surgery (RTM control) | 11 (RTM control) | 171/144 | 69 | Mean=15.8 | 6 m | +0.6 | +4% |
|  |  |  |  |  |  |  | 12 m | -0.1 | -1% |
|  |  |  |  |  |  |  | 18 m | -0.2 | -1% |
|  |  |  |  |  |  |  | 24 m | -0.2 | -1% |
|  |  |  |  |  |  |  | 30 m | -0.5 | -3% |
|  |  |  |  |  |  |  | 36 m | -0.3 | -2% |
|  |  |  |  |  |  |  | 42 m | -0.2 | -1% |
|  |  |  |  |  |  |  | 48 m | -0.1 | -1% |
| Markic 2022 Bosnia[29] | Pros (2016–2018) | Nonglaucomatous open angle | 3 (RTM control) | 31/31 | 71 | Mean=14.5 | 1 m | -1.0 | -7% |
|  |  |  |  |  |  |  | 3 m | -2.4 | -17% |
|  |  |  |  |  |  |  | 6 m | -2.2 | -15% |
| Ramli 2019 Malaysia[32] | Pros (period?) | Nonglaucomatous | 3 (RTM control) | 86/86 | 64 | Mean=16.2 | 1 w | -2.1 | -13% |
|  |  |  |  |  |  |  | 1 m | -1.8 | -11% |
| Pradhan 2012 USA[49] | Retr (2008) | 13% glaucoma, 87% nonglaucomatous; all open angle | 4 (RTM control) | 77/77 | 70 | Mean=14.8 | 1 w | -1.9 | -13% |
|  |  |  |  |  |  |  | 1 m | -2.7 | -18% |
|  |  |  |  |  |  |  | 2-3 m | -3.2 | -22% |
|  |  |  |  |  |  |  | 4-9 m | -3.2 | -22% |
|  |  |  |  |  |  |  | 10-18 m | -3.0 | -20% |
| Ngo 2016 Singapore[18] | Retr (2007–2008) | Nonglaucomatous | 1 | 116/116 | 71 | 15.4 | 1 m | -1.9 | -12% |
|  |  |  |  |  |  |  | 3 m | -2.4 | -16% |
|  |  |  |  |  |  |  | 6 m | -1.5 | -10% |
|  |  |  |  |  |  |  | 12 m | -1.3 | -8% |
|  |  | Contralateral eye (RTM control) | 1 |  |  | 15.2 | 1 m | -0.5 | -3% |
|  |  |  |  |  |  |  | 3 m | -0.9 | -5% |
|  |  |  |  |  |  |  | 6 m | -0.3 | -2% |
|  |  |  |  |  |  |  | 12 m | -0.3 | -2% |
| Park 2016 Korea[83] | Pros (2014) | Nonglaucomatous | 1 | 29/29 | 64 | 13.8 | 1 m | -1.2 | -9% |
|  |  | Contralateral eye (RTM control) | 1 |  |  | 13.4 |  | +0.3 | +2% |
| Bilak 2015 Turkey[46] | Pros (?) | Nonglaucomatous | 1 | 117/117 | 65 | 14.8 | 1 m | -2.5 | -17% |
|  |  | Contralateral eye (RTM control) | 1 |  |  | 14.5 |  | -0.2 | -1% |
| Kee 2000[91] | Pros (?) | Nonglaucomatous | 1 | 42/42 | 64 | 15.3 | 1 m | -2.4 | -16% |
|  |  | Contralateral eye (RTM control) | 1 |  |  | 15.4 |  | -0.4 | -3% |
| Yang 2013 Korea[47] | Retr (2009–2011) | Nonglaucomatous | 1 | 999/999 | 67 | 13.5 | 3 m | -1.6 | -18% |
|  |  | Contralateral eye (RTM control) | 1 |  |  | 13.6 |  | -0.2 | -1% |
| Chang 2019[92] | Pros (?) | PACG/PACS | 1 | 106/53 | 70 | 17.9 | 1 w | -4.6 | -26% |
|  |  |  |  |  |  |  | 1 m | -3.5 | -20% |
|  |  |  |  |  |  |  | 3 m | -3.5 | -20% |
|  |  | Contralateral eye (RTM control) | 1 |  |  | 16.0 | 1 w | -0.2 | -1% |
|  |  |  |  |  |  |  | 1 m | -0.2 | -1% |
|  |  |  |  |  |  |  | 3 m | -0.2 | -1% |

IOP, intraocular pressure; m, months; OHTS, Ocular Hypertension Treatment Study; PACG, primary angle-closure glaucoma; PACS, primary angle-closure suspect; POAG, primary open-angle glaucoma; preop, preoperative; pros, prospective; retr, retrospective; RTM, regression to the mean; w, weeks; y, years.
