# Supplementary material for: Evolution of intraocular pressure after cataract surgery in nonglaucomatous patients: A post-hoc analysis of PERCEPOLIS clinical trial data
Source: PLoS One. 2026 May 19;21(5):e0349310. doi: 10.1371/journal.pone.0349310 (PMC13186369; doi:10.1371/journal.pone.0349310)
Supplement: Attachment — Submitted filename: Responses to Reviewers.docx [file pone.0349310.s016.docx]

**Responses to Reviewers and the Editor**

We are very grateful to the editor and reviewers for their time and helpful comments. We have addressed all comments to the best of our ability. In particular, we conducted multiple new multivariate analyses with additional anatomical variables, and have removed the narrative review. We feel that our manuscript has been greatly improved by the revisions.

# Editor (PONE-D-25-33358)

whilst we recognise you have addressed many concerns, the concerns about the narrative review are still not resolved. We are concerned that your study, while providing some data about the literature search, does not provide sufficient information on the methodology to meet the recommendations set out by PRISMA (Preferred Reporting Items for Systematic Reviews and Meta-Analyses). Please note that we do not consider systematic review articles that do not comply with the PRISMA guidelines, including as a component of a broader study. For more information, please see <https://journals.plos.org/plosone/s/criteria-for-publication#loc-1> and <https://journals.plos.org/plosone/s/submission-guidelines#loc-systematic-reviews-and-meta-analyses>. You can find more information about the PRISMA statement here: <http://prisma-statement.org/>

We do feel however that if you remove the narrative review, or prepare it in accordance with PRISMA guidance and present the full methodology and results in the appropriate sections of your manuscript, a revised manuscript may be suitable for consideration. This would however require a substantial amount of work and thus, would be considered as a new submission.

**Reply:** To address this comment, we removed the narrative review and simply described the literature qualitatively. As a result, box plots summarizing the IOP changes in nonglaucomatous and glaucomatous eyes in the literature (previous Figs 3 and 5) were deleted and the lines graph showing IOP changes in nonglaucomatous eyes (previous Fig 4) was placed in the Supporting Information file (S1 Fig).

Moreover, the previous studies that controlled for regression to the mean (RTM) were summarized in S8 Table and depicted graphically in Fig 4 without any data synthesis (Discussion, lines 393-433). The conclusion of this literature analysis was expressed qualitatively as, “*only a small portion of the IOP-lowering effect of cataract surgery in nonglaucomatous eyes is actually due to RTM*” (lines 431, 593, and 621).

# Reviewers (PONE-D-23-26503R1)

# Reviewer #1

## Comment #1:

Regression to the mean is a statistical, not a physiological or real phenomenon. In this respect, I wonder on what basis a statement can be made about the extent “RTM does contribute to the IOP drop after cataract surgery, it accounts for only a small portion (e.g. 8–17% at 3 months). Lines 551–554”

**Reply:** To address this comment, we changed the sentence as follows:

“*our examination of the literature suggested that while RTM does contribute to the* ***perceived*** *IOP drop after cataract surgery, this contribution is small (Fig. 4)*.” (lines 593–595)

## Comment #2:

The recommendation due to possible regression to the mean phenomena was to include a control group (without treatment) because improvement in the treated group cannot be attributed only to the treatment, because even if participants are not treated, the IOP would drop simply by taking a second measurement. A treatment effect can only be shown if the IOP drops more in the treated group, compared to the control group.

**Reply:** To control for regression to the mean (RTM), it is necessary to make multiple preoperative IOP measurements over months or years before the ocular intervention: the average of these measurements then serves as the baseline measurement. Alternatively, or in addition, a cohort of patients who do not undergo the intervention could serve as a control group. Both of these controls were used in the study that most stringently addressed RTM, namely, the Ocular Hypertension Treatment Study (OHTS) on nonglaucomatous patients with ocular hypertension (ref. 48 in paper). Another control is to use the untreated fellow eye: this is a reasonable approach because the right and left eyes correlate closely in terms of IOP. These points are detailed in the “Higher preoperative IOP predicts greater falls in IOP” section in the Discussion (lines 372–420).

We did not use such controls because the primary outcome of our randomized controlled trial was endothelial cell loss after two types of cataract surgery. Consequently, we had only a single preoperative IOP measurement per study eye, and contralateral eye values were not routinely determined. Similarly, the vast majority of studies on IOP after cataract surgery have not controlled for RTM. These points are detailed in the “Higher preoperative IOP predicts greater falls in IOP” section in the Discussion (lines 387–392).

To compensate for not being able to control for RTM, we identified the sparse studies that did. They were the OHTS study (ref. 48), a similar study on probable glaucoma eyes (ref. 84), three studies that measured preoperative IOP multiple times (refs. 26, 29, 47), and six studies that used untreated fellow-eye IOP measurements (four on nonglaucomatous eyes [refs. 17, 40, 44, 45, 54] and one on PACG eyes [ref. 85]). These studies are depicted in Fig 4, summarized in S8 Table, and described in the “Higher preoperative IOP predicts greater falls in IOP” section in the Discussion (lines 393–426). The conclusion was:

“*In summary, the six studies that controlled for RTM and examined normotensive nonglaucomatous eyes suggest that cataract surgery reduces IOP in these eyes by -1.2 to -2.5 mmHg at 1–3 months, similar to the IOP changes of -2.0 and -2.4 mmHg at 1 and 3 months that we observed, respectively. By contrast, contralateral-eye IOP changes by +0.3 to -0.9 mmHg at these timepoints [17,26,29,40,44,45,54]. Thus, RTM seems to account for only a small proportion of the IOP drop after cataract surgery. This suggests that cataract surgery induces a biologically real and clinically significant change in IOP.*” (Discussion, lines 427–433)

## Comment #3:

A further recommendation (if a control group is not possible) was to adjust the analysis by baseline IOP values. Adjusting for baseline IOP means to include baseline IOP as additional explanatory variable in the statistical model. Please do not confuse this with considering the relative chance as an outcome.

**Reply:** All multivariate analyses included preoperative IOP as an explanatory variable (see Tables 4 and 5 and S4–S7 Tables).

## Comment #4:

Separate multiple regression models are not able to correctly reflect the variability that is present in the data. A model with a grouping factor (High-, Intermediate-, and Low-preoperative IOP) allows the entire (correct) variability to be used and group-specific statements to be made.

**Reply:** We chose to use separate multivariate analyses per preoperative-IOP subgroup rather than a grouping factor because our approach avoids the pitfall of a possible interaction between preoperative and postoperative IOP. This point was added to the Results section (lines 288–291).

## Comment #5:

In general, the decision not to include other anatomical variables because they might obscure the most important results is not scientifically tenable. Multivariable models are adapted for the very reason of finding the ones with a significant effect among many competing factors. Limiting the model to a few variables may exclude important ones.

**Reply:** We agree and added the three ocular anatomical variables that were available – anterior chamber depth (ACD), axial length (AXL), and lens thickness (LT) – to the multivariate analyses (see Tables 4 and 5). We also included two composite ocular anatomical variables that have been found occasionally to relate to IOP change after cataract surgery in the literature, namely, lens position (=ACD+0.5LT) and relative lens position (=[ACD+0.5LT]/AXL). Since lens position and relative lens position were collinear with ACD, AXL, and LT, they were included in separate multivariate analyses (see S6 and S7 Tables).

None of the ocular anatomical variables predicted change in IOP (see Results section, lines 260–263 and lines 302–305). The possible reasons these variables did not predict IOP change after cataract surgery are discussed in the Discussion (lines 445–510). The extensive literature on the relationship between macroanatomical variables and IOP change after cataract surgery is also newly summarized in S2 Table.

# Reviewer #2

## Comment #1:

Overall, your study presents a comprehensive analysis of the impact of cataract surgery on intraocular pressure (IOP) in non-glaucomatous patients. However, I have a few suggestions to further enhance the clarity and rigor of your work:

**Reply:** Thank you very much for the time that you have spent reviewing our manuscript, and for your helpful comments.

## Comment #2:

Discussion of Anatomical Variables: While you've provided a rationale for focusing on implant power as a representative anatomical variable, it would be beneficial to acknowledge the potential limitations of not including other ocular anatomy variables. Discussing these limitations could provide a more nuanced understanding of the study findings.

**Reply:** To address this comment, we added the three ocular anatomical variables that were available – anterior chamber depth (ACD), axial length (AXL), and lens thickness (LT) – to the multivariate analyses (see Tables 4 and 5). We also included two composite ocular anatomical variables that have been found occasionally to relate to IOP change after cataract surgery in the literature, namely, lens position (=ACD+0.5LT) and relative lens position (=[ACD+0.5LT]/AXL). Since lens position and relative lens position were collinear with ACD, AXL, and LT, they were included in separate multivariate analyses (see S6 and S7 Tables).

None of the ocular anatomical variables predicted change in IOP (see Results section, lines 260–263 and lines 302–305). The possible reasons these variables did not predict IOP change after cataract surgery are discussed in the Discussion (lines 445–510). The extensive literature on the relationship between macroanatomical variables and IOP change after cataract surgery is also newly summarized in S2 Table.

We also included the following text in the Study Limitations section:

“*Second, we did not examine other ocular macroanatomical variables because they were not routinely recorded in the trial. Of particular interest would be lens vault, iris area, and angle variables such as angle-opening distance: all have been shown to associate with IOP drop in nonglaucomatous and/or glaucomatous patients in some studies (S2 Table).*” (lines 595–598)

## Comment #3:

Statistical Analysis: You've addressed the suggestion of using a linear mixed effects model with baseline IOP as the independent variable. However, it would be helpful to provide more details on why you chose not to include these data in the paper. Explaining your decision-making process could help readers better understand the analytical approach.

**Reply:** To address this comment, we added S5 Table (which compares the standard regression analysis results to the mixed-effects modeling results) and explained in the Results section why we chose to use the standard linear regression analysis rather than the linear mixed effects model:

“*The 3-month cohort not only had the most patients (n=241) but also demonstrated the greatest reduction in IOP (-2.4 mmHg) (Fig 2). Thus, we focused on this cohort for our analyses searching for variables that associated with IOP change after cataract surgery. This approach was also supported by our preliminary multivariate analyses with both standard linear-regression multivariate analysis and linear mixed-effects modeling that used time as an independent factor: comparing these analyses showed that time had a significant effect on postoperative IOP (p=0.003) and completely abolished the significance of one independent variable (age: p changed from 0.04 in the standard analysis to 0.99 in the mixed-effects analysis) (S5 Table). This suggested that as time progresses, the mechanisms that shape postoperative IOP may change. This seems plausible. For example, while wound-healing remodeling may permit the ocular fluid to flow well at 3 months, this remodeling may have stopped at 12 months and mechanisms that impede flow have either returned, now predominate, or have newly emerged. To help interpret the data, we therefore focused on one cross-sectional timepoint, namely, 3 months*.” (lines 235–248).

The effect of time on age as an explanatory variable was also mentioned in the Discussion:

“*Notably, we observed that age disappeared as a predictive variable when time in the form of the 12-month data was included in the multivariate analysis (S5 Table), which suggests that this factor has only a transient effect on IOP after cataract surgery.*” (lines 575–577)

We also added the following limitation to the Study Limitations section:

“*Fifth, our mechanistic studies focused on only the 3-month timepoint. However, this approach was supported by the fact that the IOP drop was most pronounced at that stage. Moreover, our preliminary results suggested that IOP-shaping mechanisms may change over time (S5 Table), which could complicate data interpretation.*” (lines 603–606)

## Comment #4:

Study Limitations and Future Research Directions: While you've appropriately addressed concerns regarding the dropout rate and surgeon-related confounding variables, I encourage you to expand on the limitations of the study and suggest potential future research directions. This discussion would provide valuable insights for readers and researchers in the field.

**Reply:** To address this point, we discussed eight study limitations in the Study Limitations section (lack of RTM control, lack of other macroanatomical variables, high drop out rate at 12 months, short follow-up, focus on 3-month timepoint; single surgeon, French patients, low sample size for one subgroup analysis; lines 591–613) and expanded the Conclusions section to include:

“*These findings together suggest that different IOP-regulating mechanisms operate in even normotensive eyes. Further studies are needed to determine (i) the macroanatomical and microanatomical mechanisms by which cataract surgery lowers IOP in nonglaucomatous and glaucomatous eyes, (ii) how these mechanisms interact to shape postoperative IOP, and (iii) whether their influence changes over time after surgery.*” (lines 633–638)

## Comment #5:

Overall, your manuscript presents technically sound research with robust data analysis. The conclusions drawn are supported by the data presented, and the manuscript is written in clear and standard English. I believe that addressing the above points will further strengthen the quality and impact of your work.

**Reply:** Thank you very much for your helpful comments and time. We believe our manuscript is much improved after revising it to address your and the other reviewer’s comments.
