# Supplementary material for: Evolution of intraocular pressure after cataract surgery in nonglaucomatous patients: A post-hoc analysis of PERCEPOLIS clinical trial data
Source: PLoS One. 2026 May 19;21(5):e0349310. doi: 10.1371/journal.pone.0349310 (PMC13186369; doi:10.1371/journal.pone.0349310)
Supplement: Attachment — Submitted filename: Dear Editor Pols.docx [file pone.0349310.s017.docx]

**Dear Editor,**

Thank you for giving me the opportunity to review this manuscript. The study explores an important clinical question and is supported by a large dataset, which adds substantial value to the literature. However, despite the strength of the data, the manuscript in its current form suffers from significant issues in writing, structure, and reporting. These concerns reduce the overall clarity and scientific impact of the paper. I believe the study could make a valuable contribution if it undergoes a major revision, ideally with the assistance of an experienced scientific writer.

Below, I provide my detailed comments organised by section.

**Abstract**

•	The abstract does not provide a clear and balanced overview of the study. It lacks precise reporting of the key outcomes (mean ± standard deviation) and does not fully reflect the depth of the analysis.

•	The writing style is vague in places and does not capture the study's main strengths. A sharper, more structured abstract would enhance accessibility.

**Introduction**

•	The introduction is generally well written, but the authors rely heavily on older references when discussing mechanisms of intraocular pressure reduction after cataract surgery.

•	More recent studies using UBM and OCTA imaging should be cited, as they provide essential insights into angle parameters and trabecular meshwork morphology. Including such literature would significantly raise the relevance of the background section. *DOI:* 10.1097/IJG.0000000000001977, <https://doi.org/10.1177/1120672119879331>

*DOI:* 10.4103/meajo.MEAJO_20_19

•	Overall, the reference list is outdated and should be updated with high-impact references.

**Methods**

- The surgical description is incomplete. While it is stated that one surgeon performed 88% of procedures, the level of experience of the other surgeons is not mentioned. Were they junior or senior surgeons? Was any comparison made across surgeons? This information is essential, as surgical experience may bias the outcomes. https://doi.org/10.1007/s10792-021-02103-6
- Given the large sample size, reporting data from the primary surgeon separately would be more rigorous, which could reduce statistical bias.
- The section on tonometry raises methodological concerns. The manuscript states that if the first two readings differed, a third was taken and the mean reported. However, it is unclear what proportion of patients required a third reading. A more robust approach would be three masked measurements for all participants, performed by an experienced ophthalmologist, with the mean reported.
- For each measurement device, the **manufacturer, model, year, and country of origin** should be specified in parentheses. This is standard practice and enhances reproducibility.
- More detail is needed regarding randomisation, masking, and handling of missing data.

**Results**

- The results section mixes data reporting with interpretation. For example, lines 219–220, 224–225, 229–230, 243–248, and 280–282 contain interpretative statements that should be moved to the Discussion.
- Reporting should follow a strict descriptive format (mean ± SD, p-values with exact numbers, and confidence intervals where appropriate).
- **Tables and Figures**:
  - Table 1 appears redundant and could be removed, with its information integrated into the Introduction or Discussion.
  - Graphs are visually complex and confusing. The authors should seek input from a statistician or biostatistician to simplify the visual presentation. Improved data visualisation (clearer legends, fewer but more focused figures) would strengthen the impact.

**Discussion**

- The discussion is overly long, repetitive, and at times speculative. Key results should be summarised more concisely, and interpretation should be better distinguished from the data.
- Mechanistic explanations (e.g., regression to the mean) are relevant but present with excessive certainty. The tone should be more cautious and evidence-based.
- Readers may find it challenging to identify the central message because of repeated statements and diffuse argumentation. A sharper, more structured discussion would be beneficial.

**References**

- The reference list contains a large number of outdated studies. Replacing these with recent (last 5–7 years) peer-reviewed articles would considerably enhance the manuscript’s credibility and relevance.

**Best Regards,**

**Farshid Karimi**

BSc(Optom), MSc(Optom), PhD Candidate

School *of Optometry and Vision Science*

Faculty of Medicine and Health

Level 3, Rupert Myers Building (North Wing)

UNSW SYDNEY NSW 2052 AUSTRALIA

- +61447197463
- E: [Farshid.karimi@unsw.edu.au](mailto:email@unsw.edu.au)
