# Supplementary material for: Evolution of intraocular pressure after cataract surgery in nonglaucomatous patients: A post-hoc analysis of PERCEPOLIS clinical trial data
Source: PLoS One. 2026 May 19;21(5):e0349310. doi: 10.1371/journal.pone.0349310 (PMC13186369; doi:10.1371/journal.pone.0349310)
Supplement: Attachment — Submitted filename: Responses to Reviewers (new).docx [file pone.0349310.s018.docx]

**Responses to Reviewers (new)**

# Academic Editor:

## **Comment regarding our response to a comment from Reviewer 1**

We have sought to address all reviewer comments to the best of our ability but could not address Comment #4 of Reviewer 1. This comment requests sensitivity analyses and exploration of best/worst case scenarios. Addressing this would require significant resources and we would have to request a long extension time to be able to complete this work. We feel that this effort and resource use is disproportionate for the type of work we are presenting. We would like to emphasize that Reviewer 1 has him/herself stated that “*Analyses are generally appropriate*” and that we have thoroughly addressed all other comments from both reviewers. We have also addressed all other comments of previous reviewers, including biostatisticians, with many new analyses (see Tables 2 and 3, S5 Table, S6 Table, S8 Table, S9 Table). We hope that our paper is now acceptable for publication in *PLOS One*.

## Comment #1:

When submitting your revision, we need you to address these additional requirements.

1.Please ensure that your manuscript meets PLOS ONE's style requirements, including those for file naming. The PLOS ONE style templates can be found at

<https://journals.plos.org/plosone/s/file?id=wjVg/PLOSOne_formatting_sample_main_body.pdf> and

[https://journals.plos.org/plosone/s/file?id=ba62/PLOSOne_formatting_sample_title_authors_affiliations.pdf](https://track.editorialmanager.com/CL0/https:%2F%2Fjournals.plos.org%2Fplosone%2Fs%2Ffile%3Fid=ba62%2FPLOSOne_formatting_sample_title_authors_affiliations.pdf/1/010f0199dba43cd3-d0351679-c246-4172-9482-c9fa094269c4-000000/UWlomHPg4zKQWp6_e52LkNfuXua6LOnYi0iqyEYFcsE=232)

**Reply:** We have ensured that our manuscript is consistent with these style templates.

## Comment #2:

2. In the online submission form, you indicated that “The datasets generated during and/or analyzed during the current study are not publicly available according to French Law No. 2018-493 of June 20, 2018 on the protection of personal data (The General Data Protection Regulation (Regulation (EU) 2016/679) (GDPR: article 9) but are available from the Clinical Research Support Platform (Plateforme d’Appui à la Recherche Clinique [PARC]) of the Regional Central Hospital (CHR) of Metz-Thionville on reasonable request (email: [projetrechercheclinique@chr-metz-thionville.fr](mailto:projetrechercheclinique@chr-metz-thionville.fr), tel: +33 3 87 17 98 82). All non-archived data is subject to daily backups while all archived data is subject to duplicate storage at two different sites. This data processing is compliant with a baseline reference methodology (MR001) to which the CHR MetzThionville signed a compliance commitment on October 8, 2018.”

All PLOS journals now require all data underlying the findings described in their manuscript to be freely available to other researchers, either 1. In a public repository, 2. Within the manuscript itself, or 3. Uploaded as supplementary information.

This policy applies to all data except where public deposition would breach compliance with the protocol approved by your research ethics board. If your data cannot be made publicly available for ethical or legal reasons (e.g., public availability would compromise patient privacy), please explain your reasons on resubmission and your exemption request will be escalated for approval.

**Reply:** French law is extremely strict about patient data protection: Law No. 2018-493 on the protection of personal data explicitly states that we cannot publicly share patient data, even when anonymized. Legally, we cannot add to or change our original Data Availability statement, which is:

“*The datasets generated during and/or analyzed during the current study are not publicly available according to French Law No. 2018-493 of June 20, 2018 on the protection of personal data (The General Data Protection Regulation (Regulation (EU) 2016/679) (GDPR: article 9) but are available from the Clinical Research Support Platform (Plateforme d’Appui à la Recherche Clinique [PARC]) of the Regional Central Hospital (CHR) of Metz-Thionville on reasonable request (email:* [*projetrechercheclinique@chr-metz-thionville.fr*](mailto:projetrechercheclinique@chr-metz-thionville.fr)*, tel: +33 3 87 17 98 82). All non-archived data is subject to daily backups while all archived data is subject to duplicate storage at two different sites. This data processing is compliant with a baseline reference methodology (MR001) to which the CHR MetzThionville signed a compliance commitment on October 8, 2018.*”

## Comment #3:

3. If the reviewer comments include a recommendation to cite specific previously published works, please review and evaluate these publications to determine whether they are relevant and should be cited. There is no requirement to cite these works unless the editor has indicated otherwise. 

**Reply:** Reviewer 2 cited four papers. We did not feel that these papers pertained to our paper and therefore did not cite them.

# Reviewer #1:

As the statistical reviewer I will focus on methods and reporting. the paper is well-written and the limitations are discussed in detail by the authors.

**Comment:** Thank you very much for your time and your pertinent and helpful comments on our paper.

## Comment #1:

### Major

1) the study is now observational and needs to adhere to STROBE principles, please use an appropriate research checklist.

**Reply:** We have provided a completed STROBE checklist for cohort analyses during resubmission.

## Comment #2:

2) Analyses are generally appropriate but I would urge the authors to move away from statistical tests that only report p-values, in a cohort of limited size. Why aren't all relevant analyses conducted in a regression framework so association sizes and differences can be easily quantified, alongside their uncertainty in the form of 95% confidence intervals? these are more informative than p-values as well, and the focus should be on estimated effect sizes which should be interpreted cautiously and conservatively, considering the limited sample size (to answer this secondary research question, not the original question posed by the RCT).

**Reply:** To address this, we added effect sizes when discussing the linear mixed-modeling analysis (Lines 254-261) and the role of age (line 549-550). All other p-values in the paper were accompanied by parameters that allow the effect size to be evaluated, including partial r2 values in the regressions.

## Comment #3:

3) what do the univariable (not univariate) analyses add? I'd recommend removal (unless justification is provided).

**Reply:** Our experience with other reviewers and readers of our manuscripts indicates that many physicians like to see univariable analyses, particularly those who are less well versed in statistics: many readily understand univariable analyses but are less able to interpret multivariable analyses. Nonetheless, to address this comment, we moved the univariable analysis table (previous Table 3) to the Supplementary Materials (now S7 Table).

## Comment #4:

4) considering the levels of attrition I would expect to see sensitivity analyses using a multiple imputation framework as a mininum and even the exploration of best/worst case scenarios for the outcomes.

**Reply:** While we agree that this request is relevant, addressing it would require significant resources and we would have to request a long extension time to be able to complete this work. We feel that this effort and resource use may be disproportionate for the type of work we are presenting.

## Comment #5:

### Minor

1) multivariable not multivariate (implies numerous outcomes)

**Reply:** Thank you for this. We have made this change throughout the paper. The term “univariate” was also changed to “univariable” throughout.

## Comment #6:

2) please refrain from describing methods in the introduction, state the aims only, as you have done.

**Reply:** We have deleted the term “multivariable” from the end of the Introduction.

## Comment #7:

3) limited power is mentioned only for one subgroup analysis, it's arguably relevant for all analyses, please clarify that and rephrase to something like "power was limited and we would only be able to identify large associations with a high degree of certainty".

**Reply:** We rephrased the text as follows:

“*Finally, the sample size was limited, meaning that we would only be able to identify large associations with a high degree of certainty.*” (Lines 622-624)

## Comment #7:

I would also recommend removal of the outdated 10 subjects per covariate rule. this is so rough and depend on the type and distribution of the covariates, that is of very little use.

**Reply:** We deleted this sentence.

# Reviewer #2:

**Comment:** We would like to thank you very much for your time and your pertinent and helpful comments on our paper.

### Abstract

## Comment #1:

The abstract does not provide a clear and balanced overview of the study. It lacks precise reporting of the key outcomes (mean ± standard deviation) and does not fully reflect the depth of the analysis.

**Reply:** To address this, we expanded the Abstract to reflect other key findings and added the standard deviations for the mean IOPs as well as effect size estimates for independent variables.

## Comment #2:

The writing style is vague in places and does not capture the study's main strengths. A sharper, more structured abstract would enhance accessibility.

**Reply:** To address this, the Abstract was edited so that it transmits the key messages of our study more clearly.

### Introduction

## Comment #3:

The introduction is generally well written, but the authors rely heavily on older references when discussing mechanisms of intraocular pressure reduction after cataract surgery.

**Reply:** To address this, we changed a key reference on the potential mechanisms driving PACG and POAG (ref 3, a 2014 review) to a more recent review (Wang et al. 2024 DOI: [10.2147/EB.S472920](https://doi.org/10.2147/eb.s472920)). Another reference on this topic (ref 36) was retained because it was published in 2020.

It should be noted that despite the abundant literature on the effect of phacoemulsification on IOP in nonglaucomatous eyes, and the factors that could drive this effect, a systematic review/meta-analysis on these issues has not yet been published. Therefore, we included some older references in our bibliography to demonstrate:

(i) The consistency over time and changes in clinical practice with which IOP has been found to drop after phacoemulsification in nonglaucomatous eyes (refs 13-35; summarized in S1 Table). These studies were also used to derive S1 Fig, which shows that the change in IOP in nonglaucomatous eyes observed in our study is similar to that reported by other studies.
(ii) The current state of the literature regarding factors that shape IOP after phacoemulsification (refs 13,14,29,32–35,37–41,15,42–51,17,52–58,18,20,23–25,28) (summarized in S2 Table). While many studies have searched for such factors, the vast majority were retrospective, involved small sample sizes, examined only a few variables, conducted univariable analyses only, and/or did not exclude the use of IOP-lowering medications after surgery. Including these studies in our Introduction highlights the contribution of our study to this field, since it involves multivariable analyses of prospective data obtained from a large randomized controlled trial on patients who were naïve for IOP-lowering medication both before and after phacoemulsification surgery. Many of the previous studies were also cited when discussing our findings in the context of the literature.

## Comment #4:

More recent studies using UBM and OCTA imaging should be cited, as they provide essential insights into angle parameters and trabecular meshwork morphology. Including such literature would significantly raise the relevance of the background section. DOI: 10.1097/IJG.0000000000001977, <https://doi.org/10.1177/1120672119879331>
DOI: 10.4103/meajo.MEAJO_20_19

**Reply:** The three cited references were not directly relevant to our study because either they examined PACG/POAG patients (10.1097/IJG.0000000000001977 & [10.1177/1120672119879331](https://doi.org/10.1177/1120672119879331)) or phacoemulsification was not conducted (10.4103/meajo.MEAJO_20_19).

Notably, while reviewing these papers, we found two recent papers using LENSTAR biometry or Pentacam that assessed correlations between post-phaco change in IOP and preoperative ACD, lens thickness, change in ACD, change in iridocorneal angle, and change in anterior chamber volume (10.51329/mehdiophthal1467 & [10.3390/life15030381](https://doi.org/10.3390/life15030381)). These papers are relevant to S2 Table and thus were added to this table (refs 57 & 58) and cited in the Introduction and Discussion.

With regard to the point about UBM and OCTA, we added the following to the Study Limitations section:

“*Third, it is possible that more precise measurements of ocular macroanatomical and especially microanatomical variables, such as phase-sensitive OCT measurements of the trabecular meshwork, could find associations between these variables and IOP change.*” (Lines 593-595)

## Comment #5:

Overall, the reference list is outdated and should be updated with high-impact references.

**Reply:** Please see our reply to Comment #3.

### Methods

## Comment #6:

The surgical description is incomplete. While it is stated that one surgeon performed 88% of procedures, the level of experience of the other surgeons is not mentioned. Were they junior or senior surgeons? Was any comparison made across surgeons? This information is essential, as surgical experience may bias the outcomes. <https://doi.org/10.1007/s10792-021-02103-6>

Given the large sample size, reporting data from the primary surgeon separately would be more rigorous, which could reduce statistical bias.

**Reply:** To address this, we sought to compare the ECL in the cases conducted by the main surgeron (JMP) to the ECL in the cases conducted by the other surgeons. However, we then realized that we had excluded the cases conducted by the other surgeons for the post-hoc analysis, exactly for the reason you stated: to limit confounding due to surgeon experience. To address this, we added the following texts to the Methods and Results sections:

Methods: “*To minimize possible confounding due to differences in surgeon experience, we excluded eyes whose surgery had been conducted by surgeons other than the principal investigator (JMP).*” (Lines 170-172)

Results: “*Of the 292 patients included in the PERCEPOLIS trial, 22 (8%), 17 (6%), and 94 (32%) did not attend the 1-, 3-, and 12-month follow-up visit. Of the 270, 275, and 198 patients who attended these visits, 32, 34, and 25 were respectively excluded because IOP data were missing (n=3, 2, and 0, respectively) and/or surgery had not been conducted by the principal investigator (n=31, 32, and 25, respectively*.” (Lines 221-225)

We also altered the figure showing patient distribution in the study (Fig 1) to reflect this change.

## Comment #7:

The section on tonometry raises methodological concerns. The manuscript states that if the first two readings differed, a third was taken and the mean reported. However, it is unclear what proportion of patients required a third reading. A more robust approach would be three masked measurements for all participants, performed by an experienced ophthalmologist, with the mean reported.

**Reply:** This was the original methodology in the RCT protocol. In practice, we always take three readings and average them. To address this, we revised the Methods text as follows:

“*All IOP measurements were conducted in the same way [64] with a slit lamp-calibrated Goldmann applanation tonometer (BQ 900; year 2012; Haag-Streit, Koniz, Switzerland). Specifically, at each study visit, IOP was measured three times in immediate succession between 10:00 and 12:00. The measurement average was then recorded.*” (Lines 151-154)

## Comment #8:

For each measurement device, the manufacturer, model, year, and country of origin should be specified in parentheses. This is standard practice and enhances reproducibility.

**Reply:** To address this, we added the manufacturer, model, year of purchase, and country of origin of the Goldmann applanation tonometer (Line 152), the IOL Master (Lines 157-158), and the phacoemulsifier (Lines 146-147) to the Methods section.

## Comment #9:

More detail is needed regarding randomisation, masking, and handling of missing data.

**Reply:** The randomization and single-blinding procedures were described in detail in the original study (published in Cornea in 2021 doi:10.1097/ico.0000000000002822), as follows:

“*In June 2015 to April 2016, a convenience series of patients was examined for study eligibility during screening visits (baseline visit) in the ophthalmology department of Metz-Thionville Regional Hospital Centre (Metz, France) that involved a complete ophthalmic examination (see below). Patients were eligible for inclusion at screening if they were aged 18 years or older; their best spectacle–corrected visual acuity (BSCVA) was greater than +0.2 logMAR; they had a posterior nuclear (NO1–NO4; NC1–NC4), cortical (C1–C5), or subcapsular (P1–P5) cataract of normal to severe density, as determined by LOCS III classification25; and they con- sented to participate in the study. The exclusion criteria were white/brown cataracts; diabetes with insulin dependence/ retinal complications; preexisting cornea, ocular tone, or posterior segment pathology; preoperative endothelial cell density ,1500 cells/mm2; pregnancy; and history of retinal detachment, ocular trauma, or anterior/posterior segment surgery. Patients undergoing additional procedures apart from cataract removal and intraocular lens implantation were also excluded.*

*Eligible patients were scheduled for phacoemulsification surgery 4 to 6 weeks after screening. On arrival, they were randomly assigned 1:1 to undergo subluxation or DaC phacoemulsification by using computerized random numbers that were given to the surgeon (J.-M.P.) by the research methodologist (C.G.) in the form of sequentially numbered, opaque, sealed, and stapled envelopes. The patients were blinded to the allocation. The allocated surgical procedure was performed unless intraoperative issues caused the surgeon to convert to the other procedure*.”

We would prefer not to include these details in the present paper because they could give readers the confusing impression that our data are the primary results of a randomized clinical trial. To address this, we added the following text to the Methods:

“*The randomization and single-blinding procedures used in the RCT have been described in detail previously [62].*” (Lines 134-135)

With regard to missing data, patients with missing IOP data were excluded from the study (as indicated by Lines 169-170, 222-224). There were no missing data for the remaining variables. To address this, we added the following text to the Statistics section:

“*There were no missing data for any variables*.” (Line 192)

### Results

## Comment #10:

The results section mixes data reporting with interpretation. For example, lines 219–220, 224–225, 229–230, 243–248, and 280–282 contain interpretative statements that should be moved to the Discussion.

**Reply:** On the basis of the line numbers in the pdf of the original manuscript on the PLOS One submission site:

(i) Lines 219-220 relate to data shown in Fig. 2 and seem appropriate.
(ii) Lines 224-225, 229-230: To address this, some text was added to the Statistics section, the Results text was revised, and some text was added to the Study Limitations sections, as follows.

Statistics: “*To determine the effect of expressing IOP change as % change relative to baseline rather than as absolute IOP change in mmHg, preliminary multiple linear regression analysis with the 3-month cohort was repeated with percentage IOP change*.” (Lines 211-214)

Results: “*Postoperative change in IOP can also be defined as % IOP change relative to baseline. The % change at 1, 3, and 12 months in our cohort was -11%, -14%, and -10%, respectively. Expressing IOP as % change did not markedly alter our study outcomes: for example, our preliminary multivariable analyses showed no marked differences when we used % change rather than absolute change (S5 Table). Moreover, when we converted the IOP data in the related literature to absolute or % IOP change, we observed identical trajectories of IOP over time for both IOP variables (S1 Fig). Since absolute IOP change is generally more clinically meaningful for clinicians than % IOP change, only absolute IOP change data are shown below*.” (Lines 244-251)

Study Limitations: “*Fifth, for all analyses, we defined change in IOP as absolute change in mmHg relative to baseline, not as % IOP change. However, our preliminary multivariable analyses showed no marked differences when we used % change rather than absolute change (S5 Table). Moreover, most other studies on nonglaucomatous eyes also used this definition (S2 Table) and the few studies that compared % IOP change to absolute IOP change did not find substantial differences in the results [41,45]. Moreover, when we converted the IOP data in the related literature to absolute or % IOP change, we observed identical trajectories of IOP over time for both IOP variable types (S1 Fig)*.” (Lines 598-606)

(iii) Lines 243-248: To address this, some text was added to the Statistics section, the Results text was revised, and some text was added to the Study Limitations sections, as follows.

Statistics: “*To determine the effect of including 12-month data as well as 3-month data, we also conducted preliminary linear mixed-effects modeling with the 3- and 12-month cohorts that used time as an independent factor*.” (Lines 214-216)

Results: “*This approach was also supported by preliminary linear mixed-effects modeling that used time as an independent factor: when we compared this analysis to our standard linear-regression multivariable analysis, we found that time had a significant effect on postoperative IOP (beta=-0.72±0.25, r^2^=0.01, p=0.003), and its inclusion in the multivariable analysis completely abolished the significance of one independent variable (age: beta=0.04±0.02, r^2^=0.01, p=0.04 in the standard analysis changed to beta=0.0002±0.02, r^2^<0.001, p=0.99 in the mixed-effects analysis) (S6 Table). This suggested that as time progresses, the mechanisms that shape postoperative IOP may change. Thus, to help interpret the data, we focused on one cross-sectional timepoint, namely, 3 months*.” (Lines 254-263)

Study Limitations: “*Seventh, we focused on the 3-month data only for the multivariable analyses. However, this was supported by the fact that the IOP drop was most pronounced at that stage. Moreover, when we compared linear mixed-effects modeling that used time as an independent factor to our standard linear-regression multivariable analysis, we found that time had a significant effect on postoperative IOP and its inclusion in the multivariable analysis completely abolished the significance of age (S6 Table). This suggested that as time progresses, the mechanisms that shape postoperative IOP may change. This seems plausible. For example, while wound-healing remodeling may permit the ocular fluid to flow well at 3 months, this remodeling may have stopped at 12 months and mechanisms that impede flow have either returned, now predominate, or have newly emerged. To help interpret the data, we therefore focused on one cross-sectional timepoint, namely, 3 months*.” (Lines 607-617)

(iv) The original lines 280-282 have been deleted.

## Comment #11:

Reporting should follow a strict descriptive format (mean ± SD, p-values with exact numbers, and confidence intervals where appropriate).

**Reply:** Standard deviation values were added to all mean and beta values that were cited in our manuscript.

### Tables and Figures

## Comment #12:

Table 1 appears redundant and could be removed, with its information integrated into the Introduction or Discussion.

**Reply:** We moved Table 1 to Supplementary Materials. It is now S2 Table.

## Comment #13:

Graphs are visually complex and confusing. The authors should seek input from a statistician or biostatistician to simplify the visual presentation. Improved data visualisation (clearer legends, fewer but more focused figures) would strengthen the impact.

**Reply:** We agree that Fig 4 was confusing. Since these studies have been thoroughly reviewed in the Discussion (Lines 408-440), we moved Fig 4 to Supplementary Information. It is now S4 Fig.

### Discussion

## Comment #14:

The discussion is overly long, repetitive, and at times speculative. Key results should be summarised more concisely, and interpretation should be better distinguished from the data.

**Reply:** During revision, we substantially changed the Discussion. In particular, the regression-to-the-mean discussion was given its own section and the mechanisms sections were reorganized to improve focus, coherence, and readability.

## Comment #15:

Mechanistic explanations (e.g., regression to the mean) are relevant but present with excessive certainty. The tone should be more cautious and evidence-based.

**Reply:** We have sought to soften our language about RTM during revision.

## Comment #16:

Readers may find it challenging to identify the central message because of repeated statements and diffuse argumentation. A sharper, more structured discussion would be beneficial.

**Reply:** We have restructured the mechanisms sections so that they focus more on the subgroup analyses. We believe this has greatly improved the Discussion. We thank you for your comments.

## Comment #17:

### References

The reference list contains a large number of outdated studies. Replacing these with recent (last 5–7 years) peer-reviewed articles would considerably enhance the manuscript’s credibility and relevance.

**Reply:** Please see our reply to Comment #3.
